# Implementing machine learning methods with complex survey data: Lessons learned on the impacts of accounting sampling weights in gradient boosting

**DOI:** 10.1371/journal.pone.0280387

**Published:** 2023-01-13

**Authors:** Nathaniel MacNell, Lydia Feinstein, Jesse Wilkerson, Pӓivi M. Salo, Samantha A. Molsberry, Michael B. Fessler, Peter S. Thorne, Alison A. Motsinger-Reif, Darryl C. Zeldin

**Affiliations:** 1 Social & Scientific Systems, a DLH Holdings Company, Durham, North Carolina, United States of America; 2 Department of Epidemiology, Gillings School of Global Public Health, University of North Carolina, Chapel Hill, North Carolina, United States of America; 3 Division of Intramural Research, National Institute of Environmental Health Sciences, National Institutes of Health, Durham, North Carolina, United States of America; 4 Department of Occupational and Environmental Health, University of Iowa, College of Public Health, Iowa City, Iowa, United States of America; Vellore Institute of Technology: VIT University, INDIA

## Abstract

Despite the prominent use of complex survey data and the growing popularity of machine learning methods in epidemiologic research, few machine learning software implementations offer options for handling complex samples. A major challenge impeding the broader incorporation of machine learning into epidemiologic research is incomplete guidance for analyzing complex survey data, including the importance of sampling weights for valid prediction in target populations. Using data from 15, 820 participants in the 1988–1994 National Health and Nutrition Examination Survey cohort, we determined whether ignoring weights in gradient boosting models of all-cause mortality affected prediction, as measured by the F1 score and corresponding 95% confidence intervals. In simulations, we additionally assessed the impact of sample size, weight variability, predictor strength, and model dimensionality. In the National Health and Nutrition Examination Survey data, unweighted model performance was inflated compared to the weighted model (F1 score 81.9% [95% confidence interval: 81.2%, 82.7%] vs 77.4% [95% confidence interval: 76.1%, 78.6%]). However, the error was mitigated if the F1 score was subsequently recalculated with observed outcomes from the weighted dataset (F1: 77.0%; 95% confidence interval: 75.7%, 78.4%). In simulations, this finding held in the largest sample size (N = 10,000) under all analytic conditions assessed. For sample sizes <5,000, sampling weights had little impact in simulations that more closely resembled a simple random sample (low weight variability) or in models with strong predictors, but findings were inconsistent under other analytic scenarios. Failing to account for sampling weights in gradient boosting models may limit generalizability for data from complex surveys, dependent on sample size and other analytic properties. In the absence of software for configuring weighted algorithms, post-hoc re-calculations of unweighted model performance using weighted observed outcomes may more accurately reflect model prediction in target populations than ignoring weights entirely.

## Introduction

Machine learning is rapidly gaining traction in epidemiology and related disciplines [1–10], but several challenges continue to impede its widespread integration into the field [1,11]. One challenge that has received little attention is how to use machine learning to analyze data from complex surveys such as the National Health and Nutrition Examination Survey (NHANES).

NHANES and other national surveys have a long history in epidemiology and are widely utilized for research purposes, national surveillance initiatives, and clinical reference values (e.g., growth standards) [12,13]. Their prominence stems in large part from their population-based sampling designs, which allow estimates computed from these data to be generalized to target populations. However, the generalizability of results from complex survey data to target populations hinges on utilizing appropriate analytic methods. Such methods include the use of sampling weights as well as the incorporation of design variables that account for any departures from simple random sampling, including differential selection probabilities, stratification, and geographic clustering [14]. Many currently available software packages for implementing popular machine learning procedures have given inadequate consideration to the implications of using these models to analyze complex survey data. Unlike conventional statistical software packages, which are well-equipped to account complex survey designs, machine learning software implementations offer no or limited options for handling complex samples. For example, the documentation for the popular ‘SuperLearner’ R package states that the optional observation weights are passed to the algorithms, but many of the built-in wrappers ignore or are unable to use the provided weights [15].

Little is known about how ignoring complex survey design elements, including sampling weights, in machine learning analyses affects the generalizability of conclusions drawn from the algorithms to target populations. We assessed whether specifically accounting for survey sampling weights affects the performance of gradient boosting, a powerful ensemble classification algorithm. We first conducted a ‘real world’ case study using NHANES III data to predict all-cause mortality, assessing the impact of incorporating the survey weights in various stages of the model configuration and evaluation process. We then conducted a series of simulations to determine if the impact of accounting for the weights varies under certain theoretical scenarios for sample size, predictor strength, survey weight variability, and model dimensionality. Our study is one of the few that has investigated the implications of implementing machine learning methods with complex survey data. The study findings highlight the importance of implementing weighted algorithms and provide novel information on how recalculating model performance post-hoc with weighted outcome data may offer practical advantages in the absence of software for configuring weighted algorithms.

## Materials and methods

### Case study

The NHANES III (1988–1994) is a population-based survey that utilized a complex, multistage, stratified probability sampling design to select participants representative of the civilian, non-institutionalized United States (U.S.) population [16,17]. Mortality status was ascertained via linkage to the Public-use Linked Mortality Files, which includes vital statistics based on the National Death Index for survey participants ≥18 years of age from the date of survey participation through December 31, 2015 [18]. All adults ≥18 years of age who completed both the household interview and physical examination components of the study were eligible for inclusion in the present analysis (N = 17,752). We further excluded 13 individuals with insufficient identifiers to confirm their mortality status in the National Death Index and 1,919 individuals with incomplete predictor data, resulting in a final sample size of 15,820. Average follow-up time for included participants was 21.0 years (deceased participants: 12.9 years; alive participants: 24.0 years). Sampling weights in NHANES III were calculated using a three-stage approach (individuals nested within households, nested within primary sampling units), starting with a base weight that accounts for the survey’s targeted over-sampling of hard-to-reach demographic groups. This base-weight was then updated for observed non-response and post-stratified to match the U.S. non-institutionalized population demographic structure estimated by the U.S. Census. The sampling weights in the analytic dataset ranged from 221–132,279, with a mean of 9,565 (standard deviation = 11,820). The NHANES III received National Center for Health Statistics Research Ethics Institutional Review Board approval and written informed consent was obtained from all participants.

We selected 27 predictor variables from the NHANES III household interview and physical examination datasets based on their suspected association with all-cause mortality. All predictors were assessed at study enrolment and included sociodemographic characteristics [age (continuous), sex (male, female), race/ethnicity (non-Hispanic White, non-Hispanic Black, Mexican American, other), educational attainment (less than high school; high school, some college, or associate’s degree; college degree or above), census region (Northeast, Midwest, South, West), urbanicity (metro, rural), household income (above or below $20,000 per year), marital status (married, unmarried), country of origin (born in the U.S. or not), insurance coverage (insured, uninsured), health behaviors (tobacco smoke exposure (current smoker, former smoker, second-hand exposure, not exposed), self-rated general health status (excellent, very good, good, fair, poor), use of vitamin/mineral supplements (yes, no), use of prescription medicines (yes, no), and clinical characteristics (body mass index (continuous), systolic and diastolic blood pressure (continuous), history of congestive heart failure (yes, no), stroke (yes, no), asthma (yes, no), non-skin cancer (yes, no), diabetes (yes, no), hypertension (yes, no), heart attack (yes, no), or chronic bronchitis or emphysema (yes, no), hospitalization in past year (yes, no), five or more doctor visits in past year (yes, no)]. Each of these covariates was measured using the NHANES III questionnaire, excepting body mass index and systolic and diastolic blood pressure, which were measured during examination, and all analyses were adjusted for this set of predictor variables as described.

### Simulations

We created semi-synthetic simulated datasets by first resampling from our NHANES III analytic dataset. Participants were sampled randomly with replacement, with observations allocated into strata proportional to their distribution in the NHANES III data structure. To evaluate whether the impact of accounting for standard NHANES III sampling weights is influenced by sample size, we created five resampled datasets of various sizes (i.e., n = 10,000; 5,000; 2,500; 500; 250). For each resampled dataset, we simulated the response variable using logistic regression with β coefficients corresponding to marginal associations observed in the real NHANES III sample.

We then created additional modifications to these five ‘baseline’ simulations to determine whether the importance of accounting for sampling weights was affected by stronger vs. weaker predictor strength (predictor β coefficients doubled and halved, respectively), increased vs. reduced sampling weight variability (changes to the sampling weight variance was achieved by simulating sampling weight values from a log-normal distribution with mean equal to the mean weight of the NHANES III sample and variance doubled and halved, respectively, representing the degree of departure from a simple random sample), and reduced dimensionality (predictor β coefficients comparable to NHANES III for a subset of 10 predictors and coefficients set to zero for the remainder). In the case of the latter simulation, a subset of the categorical variables and a subset of the continuous variables were chosen to be informative in the reduced dimensionality simulations at a comparable ratio to the full variable set. Selection within the categorical and continuous variable sets were chosen at random. As a sensitivity analysis, we also created a null simulation scenario in which all predictor β coefficients were set to zero, for additional reassurance that a model without predictors performed as expected (no better than chance). The resulting 35 simulations are summarized in **S1 and S2 Tables**.

### Gradient boosting approach

We fit gradient boosting models with the *xgboost* 1.3.0 package in Python Version 3.6 (Python Software Foundation, Beaverton, Oregon). Classification trees identify a sequence of data splits by repeatedly identifying which variable can best divide the data into groups that maximize within-group homogeneity in the outcome relative to the previous classification system. The *xgboost* ensemble approach adds additional classification trees in an attempt to improve prior predictions (gradient boosting). This process produces an ensemble model that can be thought of as a set of regression models, each individually tuned to minimize prediction error for different population strata and for different “mechanisms” (i.e., interactions between predictors) within these strata identified as informative for the outcome by the algorithm. In the binary case applied here, the final predicted probabilities are calculated by averaging the individual tree linear predictors. For a more in-depth introduction to gradient boosting, we refer the reader to the original papers by Friedman [19,20] and an applied example by Zhang et al [21]. We evaluated model performance using the F1 score. The F1 score is a percentage value and is used to represent the harmonic mean of the models’ sensitivity and positive predictive value. We chose the F1 score to balance the importance of detecting cases versus non-cases in a single score for our scenario, in which cases are rare (i.e., to represent a clinically-relevant prediction algorithm). In prioritizing this balance, the F1 score was preferable over simpler performance metrics like log-loss, which would undesirably emphasize detecting non-cases because they are more common. Further information on the calculation and use of the F1 score is provided in **S1 Appendix**.

To improve model performance while preventing over-fitting, the gradient boosting configuration process requires tuning many learning (number of trees, tree depth, growth rate) and regularization (observation and variable bagging, stopping criteria, and number-of-variable L1/L2 penalties) hyper-parameters. To tune the hyper-parameters, we performed a randomized search of the hyper-parameter space in a massively parallelized computing environment (using approximately 151,140 central processing unit hours) to identify hyper-parameter configurations yielding the best predictions [22]. A brief description of these hyper-parameters and their search space ranges is provided in **S2 Appendix**. We used the *RandomizedSearchCV* function in the Python library *SciKitLearn* version 0.24.1 [23] within the National Institutes of Health High-Performance Computing Biowulf cluster (http://hpc.nih.gov) to test 500, 000 combinations of 11 hyper-parameter sets for each simulation. To do so, we randomly sampled from a uniform distribution covering reasonable ranges for each parameter because the hyperparameter space was too large for a grid search.

We evaluated each model implementation using five-fold cross-validation and a custom python script that enabled us to control how weights were used in the *xgboost* model. To validate our custom script, we re-calculated F1 scores of the final model fits using the *MetricsWeighted* package version 0.5.1 in R 4.0 (R Core Team, Vienna, Austria). To calculate performance metrics for each model, we used beta parameters derived from the out-of-fold data for our subsequent evaluation of the survey weights, so that the prediction model used for each observation did not use data from that observation itself. To give a sense of the sensitivity of these metrics to changes in the underlying data, we calculated 95% percentile confidence intervals (CI) bootstrapped at the predicted outcome probability level using the *boot* R package.

To qualitatively gauge the impact of our model configuration process on our findings, we performed sensitivity analyses by fitting each model with the default hyper-parameters settings. The final values for the 11 hyper-parameters utilized in each simulation are shown in **S1 Table** (weighted models) and **S2 Table** (unweighted models), along with the range of values searched for each and the default values at the time.

### Evaluation of survey weights

To demonstrate gradient boosting’s real-world and theoretical performance in the context of survey weights, we used the predicted probabilities generated from the optimized gradient boosting model configurations to calculate F1 scores under three scenarios. In Scenario One, which represents the “gold standard” implementation, calculated F1 scores were weighted by the distribution of observed outcomes (to estimate ‘real-world’ performance in the U.S. population) and predicted outcomes were based on the predicted probabilities from the weighted gradient boosting model configuration (to capture the performance of a model ‘aware’ of the weights). Scenario One represents the valid approach for incorporating survey weights in traditional epidemiologic analyses to obtain results that generalize to the target population. In Scenario Two, observed outcomes were based on the unweighted data (to capture ‘crude’ predictive performance in the sample), and predicted outcomes were based on the predicted probabilities from the unweighted gradient boosting model configuration (to capture the performance of a ‘crude’ model only aware of the sample). Scenario Two is included in these analyses as an example of an invalid use of complex survey data; ignoring the survey weights in the analyses does not account for differences in the distribution of effect modifiers between the study sample and target populations and, traditionally, produces results that do not generalize to the target population. While the F1 scores calculated for Scenarios One and Two are equivalent to the fully optimized versions of the models configured with and without weights output by the software packages we used, Scenario Three involved a modified approach. In Scenario Three, F1 scores were calculated using assumed observed outcomes based on the *weighted* data and predicted outcomes (‘real-world’ performance) where the predicted probabilities were estimated from the *unweighted* gradient boosting model configuration (a model without awareness of the sample design). Scenario Three demonstrates the real-world effect of ignoring “true” weights when selecting the best learner and determines whether prediction based on models that are configured naïve to weights can be generalized to target populations if the final configured model is scored in appropriately weighted data (for instance, used to predict outcomes in a new, representative sample). To illustrate the potential error resulting from ignoring sampling weights, we calculated the difference between the F1 scores for Scenarios Two and Three compared to the F1 score for the “gold standard” Scenario One. We performed this comparison using the NHANES III dataset and repeated it for each simulation. Example Python code is provided in the **S1 Code**.

## Results

### Case study–NHANES III

The distribution of all predictors included in the gradient boosting models are shown in **Table 1**, stratified by mortality status. Compared to those who remained alive, deceased participants were older and less likely to be female. They were also less educated, poorer, and more likely to report being a former smoker, having poor health, and experiencing adverse health conditions.

**Table 1:** Descriptive characteristics of the National Health and Nutrition Examination Survey III analytic population^a^.

	Overall	Alive	Deceased
	(N = 15,820)	(N = 9,683)	(N = 6,137)
Median (interquartile range)			
Age (years)	40 (29–56)	35 (27–44)	64 (53–73)
Body mass index (kg/m^2^)	25 (22–29)	25 (22–29)	27 (23–30)
Systolic blood pressure (mmHg)	118 (109–130)	114 (107–124)	133 (120–147)
Diastolic blood pressure (mmHg)	73 (67–80)	73 (66–79)	75 (68–82)
N (%)			
Female	8,482 (53)	5,522 (54)	2,960 (50)
Race/Ethnicity			
Non-Hispanic White	6,792 (77)	3,451 (75)	3,341 (81)
Non-Hispanic Black	4,435 (11)	2,974 (11)	1,461 (11)
Mexican American	3,986 (5)	2,807 (5)	1,179 (4)
Other	607 (7)	451 (8)	156 (5)
Education			
Less than HS	6,340 (25)	3,002 (19)	3,338 (40)
HS/Some College/AD	7,519 (55)	5,282 (58)	2,237 (48)
College Degree or Above	1,961 (20)	1,399 (23)	562 (13)
Census region			
Northeast	2,219 (20)	1,321 (20)	898 (20)
Midwest	3,187 (25)	1,897 (25)	1,290 (26)
South	6,798 (34)	4,077 (34)	2,721 (35)
West	3,616 (21)	2,388 (21)	1,228 (20)
Urbanicity (metro)	7,747 (48)	5,130 (50)	2,617 (44)
Annual household income <$20K (yes)	7,605 (32)	3,935 (27)	3,670 (47)
Married (yes)	9,363 (65)	5,875 (66)	3,488 (62)
Born in U.S. (yes)	12,765 (87)	7,499 (86)	5,266 (91)
Insurance coverage (yes)	13,146 (87)	7,534 (85)	5,612 (93)
Tobacco smoke exposure			
Current smoker	3,936 (28)	2,474 (28)	1,462 (28)
Former smoker	3,896 (25)	1,816 (21)	2,080 (35)
Secondhand	1,381 (7)	1,018 (8)	363 (4)
Not exposed	6,607 (40)	4,375 (43)	2,232 (33)
General health			
Excellent	2,450 (21)	1,850 (24)	600 (12)
Very good	3,844 (32)	2,684 (35)	1,160 (23)
Good	5,675 (32)	3,539 (31)	2,136 (36)
Fair	3,117 (12)	1,443 (9)	1,674 (22)
Poor	734 (3)	167 (1)	567 (7)
Congestive heart failure (yes)	569 (2)	64 (0.4)	505 (6)
Stroke (yes)	482 (2)	51 (0.4)	431 (6)
Asthma (yes)	1,086 (8)	635 (8)	451 (8)
Non-skin cancer (yes)	627 (4)	148 (2)	479 (8)
Diabetes (yes)	1,267 (5)	310 (2)	957 (13)
Hypertension (yes)	4,340 (23)	1,695 (16)	2,645 (42)
Heart attack (yes)	735 (3)	87 (0.9)	648 (10)
Chronic bronchitis/emphysema (yes)	1,121 (8)	390 (5)	731 (14)
Supplement use (yes)	5,997 (42)	3,458 (41)	2,539 (45)
Prescription medication use (yes)	7,377 (45)	3,298 (36)	4,079 (67)
Hospitalization in past year (yes)	2,301 (12)	1,113 (10)	1,188 (17)
5+ doctor visits in past year (yes)	3,769 (22)	1,822 (19)	1,947 (31)

^a^ Medians and percents represent weighted distributions; frequencies are unweighted.

The F1 scores for each gradient boosting model implementation in the NHANES III data are shown in **Table 2**. Scenario One, the “gold standard” weighted model, resulted in an F1 score of 77.4% (95% CI: 76.1%, 78.6%). Compared to the gold standard implementation, masking the weights (Scenario Two) increased the F1 score by five percentage points (81.9%; 95% CI: 81.2%, 82.7%). The F1 score for Scenario Three, which masked weights during the model configuration process, but incorporated the weights during the final model re-evaluation step, performed similarly (77.0%; 95% CI: 75.7%, 78.4%) to the gold standard. F1 scores estimated under each design scenario are provided in **S3** and **S4 Tables.**

**Table 2:** Performance of unweighted versus weighted gradient boosting model implementations using data from the National Health and Nutrition Examination Survey III.

Scenario	Model configuration	Model evaluation	F1 Score (%)	Error^a^
One	Weighted	Weighted	77	REF
Two	Unweighted	Unweighted	82	+5
Three	Unweighted	Weighted	77	-0

^a^ Difference in F1 score compared to model trained and scored with weighted data. All displayed values were rounded to the 3^rd^ decimal place after errors were calculated from unrounded F1 scores. Displayed errors may therefore be nominally different than would be expected if calculated from the displayed F1 scores.

### Simulation study

**Fig 1:**
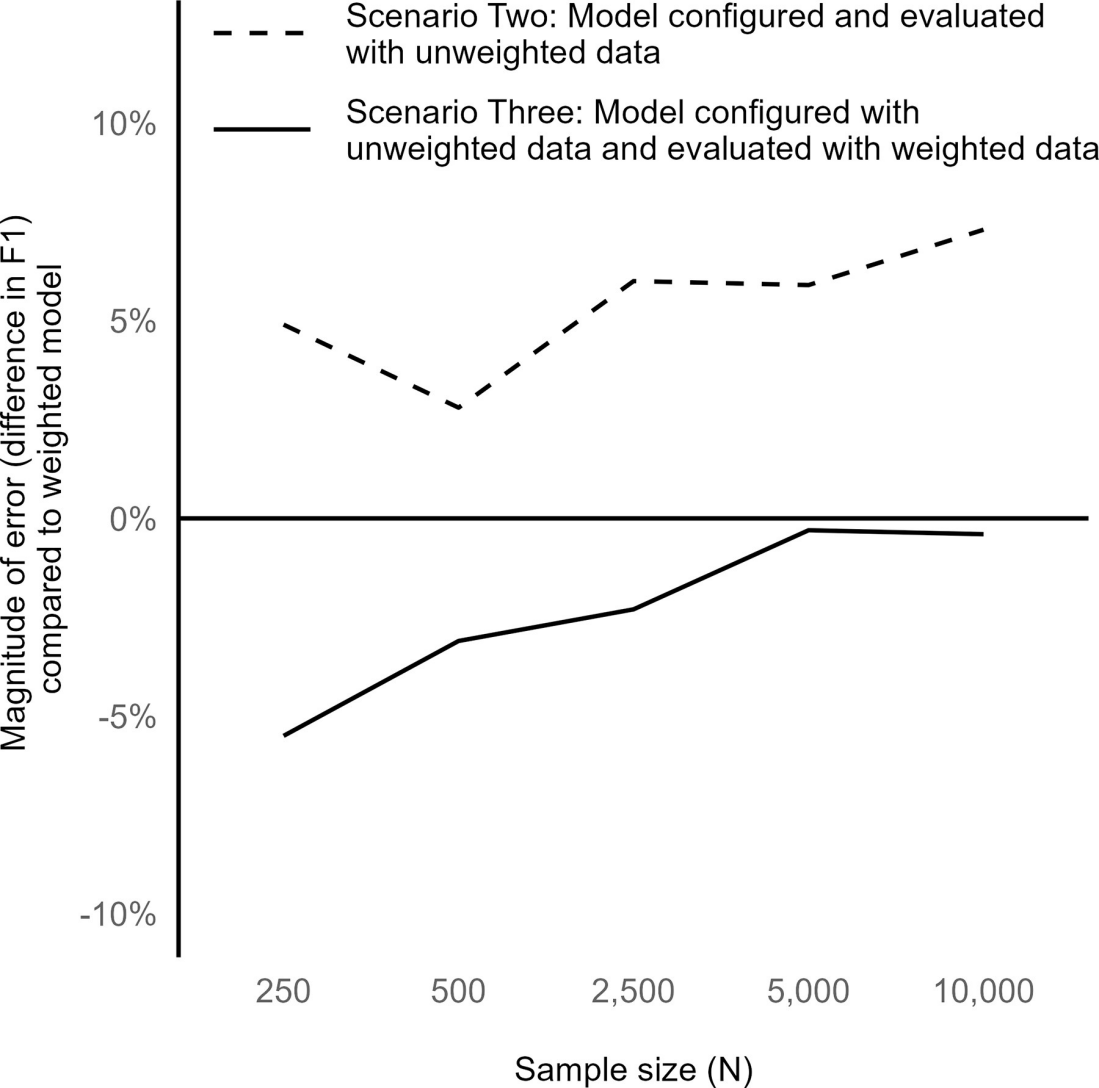
Performance of unweighted versus weighted gradient boosting model implementations by sample size (baseline simulations).

The error in the F1 score when comparing Scenarios Two and Three to gold standard Scenario One, with variation by sample size is shown in **Fig 1**. Scenario Two resulted in an increased F1 score compared to Scenario One, with a slightly higher error at sample sizes of 2,500–10,000 compared to sample sizes of 250–500. The error for Scenario Three was the greatest for small sample sizes (N = 250, 500, and 2,500) and approached zero for larger sample sizes (N = 5,000 and 10,000). At a sample size of 10,000, Scenario Three resulted in F1 scores comparable to Scenario One regardless of the other analytic factors that we varied (weight variability, predictor strength, dimensionality) (**Table 3**).

**Table 3:** Performance of gradient boosting models configured and evaluated under different analytic scenarios, holding sample size constant at N = 10,000.

	F1 Score (%)			F1 Score (%)	
Study design feature	Scenario One^a^ (gold standard)	Scenario Two^b^	Error^d^^,^^f^	Scenario Three^c^	Error^e^^,^^f^
High variability in weights	81	79	-2	80	-1
Low variability in weights	80	80	-0	80	-0
Strong marginal predictors	85	89	+4	84	-1
Weak marginal predictors	58	66	+9	58	+1
Fewer marginal predictors (10)	68	74	+6	68	+1
No marginal predictors	32	28	-4	25	-7

NHANES III, National Health and Nutrition Examination Survey III.

^a^ Scenario One: Gradient boosting model configured and evaluated on weighted data (gold standard model).

^b^ Scenario Two: Gradient boosting model configured and evaluated on unweighted data.

^c^ Scenario Three: Gradient boosting model configured on unweighted data and evaluated on weighted data.

^d^ Difference in F1 score for Scenario Two compared to Scenario One.

^e^ Difference in F1 score for Scenario Three compared to Scenario One.

^f^ All displayed values were rounded to the 3^rd^ decimal place after errors were calculated from unrounded F1 scores. Displayed errors may therefore be nominally different than would be expected if calculated from the displayed F1 scores.

**Fig 2:**
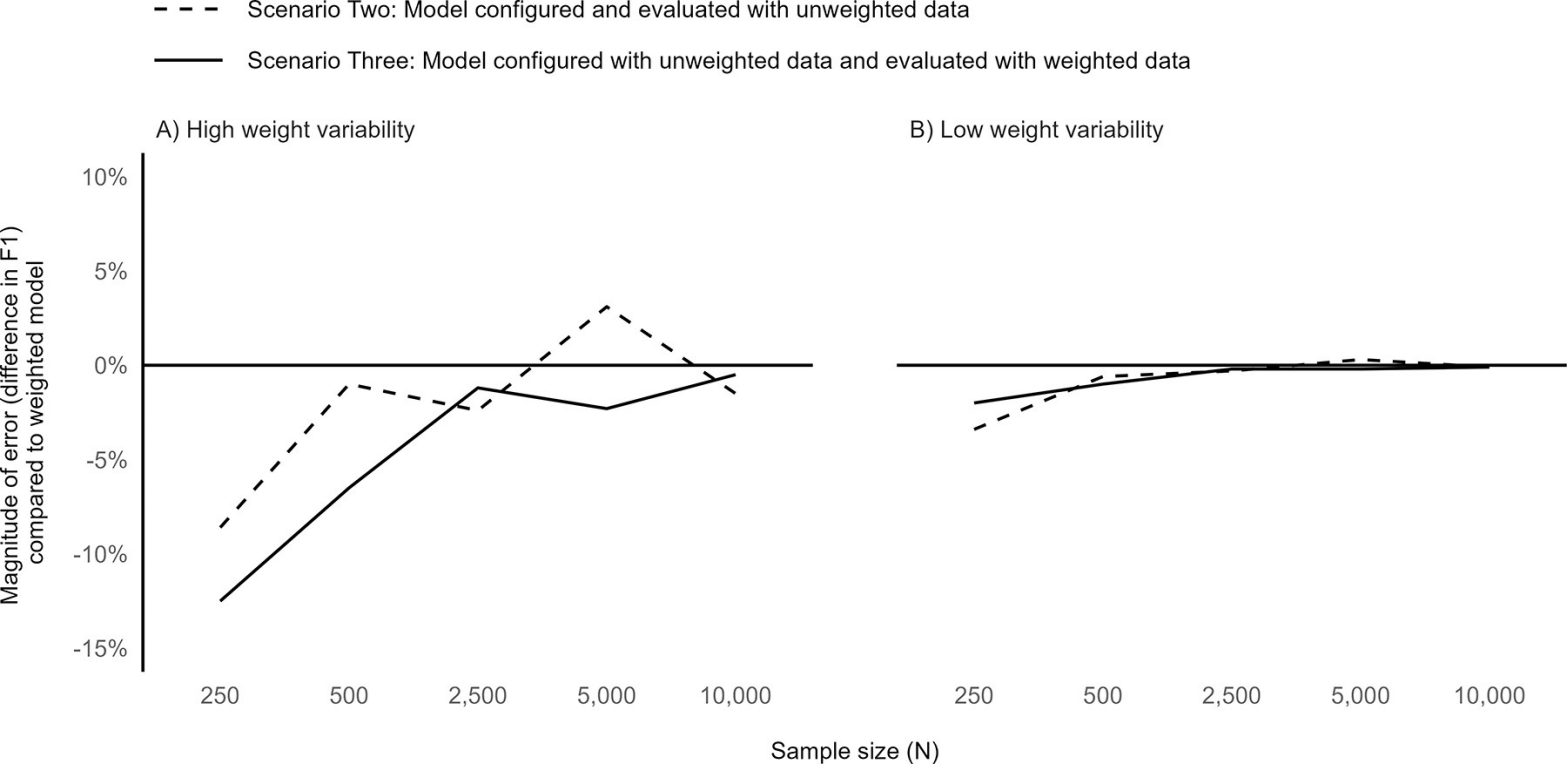
Performance of unweighted versus weighted gradient boosting model implementations by weight variability and sample size.

Compared to the baseline simulation model results (**Fig 1**), variation by sample size differed in simulations with higher or lower sampling weight variability between the observations. When the weight variability was higher (**Fig 2A**), Scenarios Two and Three resulted in similarly biased F1 scores at low sample sizes and similarly unbiased F1 scores at high sample sizes. When weight variability was low (**Fig 2B**), accounting for the weights during either the model configuration process or evaluation step had little impact.

**Fig 3:**
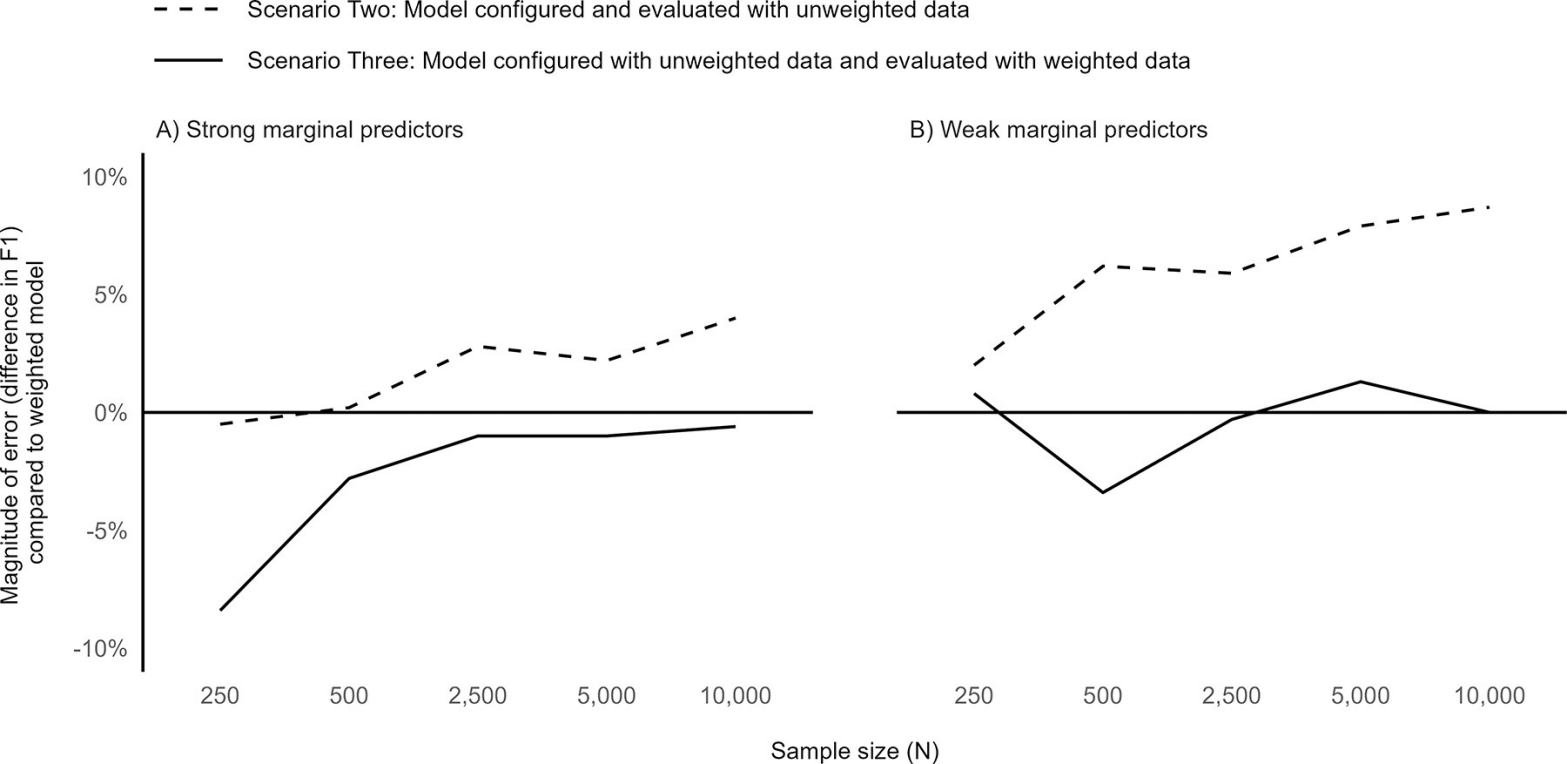
Performance of unweighted versus weighted gradient boosting model implementations by predictor strength and sample size.

Varying the predictor strength (**Fig 3**) did not alter the overall trends we observed by sample size in the baseline simulation model (**Fig 1**). Regardless of whether the marginal predictor strength was stronger (**Fig 3A**) or weaker (**Fig 3B**), error in the F1 score remained even at high sample sizes in Scenario Two, but largely diminished at high sample sizes in Scenario Three. However, we observed an overall reduction in F1 score errors across all sample sizes for Scenario Two in models with strong marginal predictors compared to those with weak marginal predictors. Additionally, with weak marginal predictors, Scenario Three resulted in little F1 score error even at small sample sizes. The models with fewer predictor variables performed similarly to the baseline simulation models (**S1 Fig**). A comprehensive list of the F1 scores for all simulations is available in **S3 Table**.

### Sensitivity analyses

In null models that contained no marginal predictors, the F1 score was low for all Scenarios (**S3 Table**) and biased for Scenarios Two and Three compared to Scenario One for all sample sizes (**S2 Fig**). Results for the model implementations using the default hyper-parameters are shown in **S4 Table**. We saw similar but less consistent patterns compared to the primary model implementations. The default model runs had a larger average departure of Scenarios Two and Three from the gold standard Scenario One, with particularly large errors for simulations in small samples.

## Discussion

Our findings suggest that failing to account for sampling weights in gradient boosting models of complex survey data may affect model performance in target populations, even at relatively large sample sizes. However, depending on the study design and other analytic factors, weighting may be less important to the model configuration process than might be expected in a traditional regression model. This partial correction for lack of weights may occur because the algorithm has access to covariates that are (at least partially) correlated with the design strata. As such, even if the model does not have access to the actual design weights, it may be able to partially recover the design structure through selection of appropriate coefficient and interaction terms (i.e., post-stratification) in place of direct weighting if model performance can be scored in “real-world” (weighted) data. In the NHANES III dataset, we found that model performance evaluated using observed outcomes from the weighted dataset was comparable regardless of whether predicted probabilities were generated from weighted or unweighted models. At the largest sample size assessed (N = 10,000), this finding held across the other analytic factors simulated, including various scenarios for weight variability, predictor strength, and model dimensionality.

At small sample sizes, whether re-evaluating the models with weighted observed outcomes mitigated the error from models configured with unweighted observed outcomes depended on other analytic factors. Accounting for the sampling weights in either the model configuration process or re-evaluation step had little impact when the variability in sampling weights was low, suggesting that weights can potentially be ignored if there is little deviation from a simple random sample. Additionally, our findings suggest that the impact of ignoring the weights entirely may be negligible in models with particularly strong marginal predictors. Neither condition was met in our baseline simulations mimicking the NHANES III study design. In these simulations, the error associated with failing to account for the sampling weights during the model configuration process was not mitigated by incorporating the weights in the re-evaluation step at smaller sample sizes as it was at the largest sample size (N = 10,000).

To our knowledge, only one other paper has examined the implications of implementing machine learning methods with complex survey data [24]. In their study, Toth and Eltinge assessed the effects of ignoring sampling weights when applying a recursive partitioning algorithm and concluded that the weighted estimator was substantially less biased than the unweighted estimator. However, the authors’ analysis focused on a simple, single-tree algorithm. Our findings provide evidence that their conclusion may also extend to more flexible ensemble recursive partitioning algorithms such as gradient boosting, but not necessarily under all study conditions. Toth and Eltinge also found that the asymptotic consistency of predictions derived from weighted recursive partitioning algorithms may fail in the case of extreme clustering relative to small sample sizes, suggesting that tree models may split small data into clusters and effectively ‘memorize’ the study design structure instead of learning the underlying population’s joint variable distributions. While the goal of the present paper was to evaluate the effect of failing to account for sampling weights in gradient boosting models, more in-depth investigations of other survey design elements—such as stratification and geographic clustering—in the context of ensemble and other machine learning algorithms are warranted.

Our findings may be specific to the evaluation metric we used, and it should be noted that there is currently no consensus regarding the most appropriate evaluation metric for unbalanced data. We selected the F1 score because it and the related precision-recall plot have been shown to have advantages compared to the area under the curve (AUC) in the presence of class imbalance [25,26] although the Mathews correlation coefficient may offer additional advantages [27]. Evaluating other performance metrics was beyond the scope of this proof-of-concept analysis and is another avenue for future research.

The flexibility of gradient boosting requires carefully tuning many hyper-parameters that represent scaling factors for various components of the model growth and regularization process [3,28–30]. A strength of our analysis is our model configuration approach, which optimizes the hyper-parameter set using a randomized, multidimensional grid search [22]. Although computationally intensive, we prioritized a more comprehensive model tuning approach to mitigate the chance of generating spurious findings due to likely differences between the optimal hyper-parameter set for the various model implementations. For the same reason, our findings may be specific to our hyper-parameter optimization approach and may not hold if little effort is made to appropriately tune the models. Indeed, our sensitivity analysis using the default hyperparameter settings for all model implementations produced less consistent results and generally performed poorer, underscoring the importance of tuning when implementing gradient boosting.

Our study is not without limitations. We chose to use gradient boosting as a test case because of its computational efficiency and good performance relative to other tree-based algorithms [31,32]. This approach shows good real-world performance in data mining competitions such as Kaggle [33], but the search for optimal model hyperparameters can be computationally intensive and unoptimized models can be prone to overfitting [29]. To mitigate potential overfitting, we explicitly tuned the models’ regularization hyperparameters and utilized five-fold cross-validation. Although five folds are commonly used for cross-validation, some have called for additional cross-folds for gradient boosting, particularly when used with small sample sizes [28]. We considered additional folds but ultimately prioritized increased computational efficiency, which was a major challenge we faced in implementing our analysis given the numerous simulations and tens of millions of individual model runs involved. Results from our null models, which yielded uniformly poor F1 scores, offered some reassurance concerning potential overfitting, as did our observation that F1 scores increased with increasing predictor strength. In clinical and other practical applications of gradient boosting, using more than five folds for cross-validation should be strongly considered.

Our experience implementing this study and its key findings indicate that epidemiologic research may benefit from more readily available (and validated) software options for weighting machine learning analyses of complex survey data, along with clear tutorials for model configuration. In the absence of accessible software packages for implementing weighted algorithms, post-hoc calculation of performance metrics on weighted data may represent the best alternative for the average user when generalizability is a priority. However, such results should be interpreted with caution, particularly at small sample sizes, and future studies should be conducted to confirm our findings across a range of algorithms. NHANES and other national surveys continue to play an important role in epidemiologic research. As the popularity of machine learning increases, failure to give more attention to appropriately analyzing complex survey data may represent a missed opportunity to leverage these powerful approaches.

## Conclusions

Failing to account for sampling weights in gradient boosting models of complex survey data affects prediction, dependent on sample size and other analytic properties. In the absence of software for configuring weighted algorithms, our findings indicate that post-hoc re-calculations of unweighted model performance using weighted outcome data may produce more accurate predictions for the target population and therefore produce less biased results than ignoring survey weights entirely. Additional research is warranted to confirm these results for gradient boosting models. While outside the scope of this work, further investigation is also needed to expand our understanding on how to appropriately use and implement other machine-learning algorithms, including deep learning methods, with complex survey data. In addition to post-hoc recalculation approaches such as ours, such investigations may give consideration to techniques for directly incorporating weights into the analyses [34,35] and model explanation methods (e.g., Shapley additive explanations) depending on the analytic method and software availability.

## Supporting information

S1 Fig Performance of unweighted versus weighted gradient boosting model implementations with fewer marginal predictors (10), by sample size. (TIF)

S2 Fig Performance of unweighted versus weighted gradient boosting model implementations with no marginal predictors, by sample size. (TIF)

S1 Table Description of case study and simulations performed, and final hyper-parameter sets for weighted models. (DOCX)

S2 Table Description of case study and simulations performed, and final hyper-parameter sets for unweighted models. (DOCX)

S3 Table Performance of gradient boosting models configured and evaluated under different design scenarios. (DOCX)

S4 Table Performance of gradient boosting models run with default hyper-parameters under different design scenarios. (DOCX)

S1 Appendix Using F1 score as an evaluation metric. (DOCX)

S2 Appendix XGBoost hyperparameter interpretations and ranges used for hyperparameter sample space. (DOCX)

S1 Code Example code to create simulated datasets, configure gradient boosted model, and calculate performance metrics. (PDF)
