# Supplementary figures and images for: Implementing machine learning methods with complex survey data: Lessons learned on the impacts of accounting sampling weights in gradient boosting
Source: PLoS One. 2023 Jan 13;18(1):e0280387. doi: 10.1371/journal.pone.0280387 (PMC9838837; doi:10.1371/journal.pone.0280387)

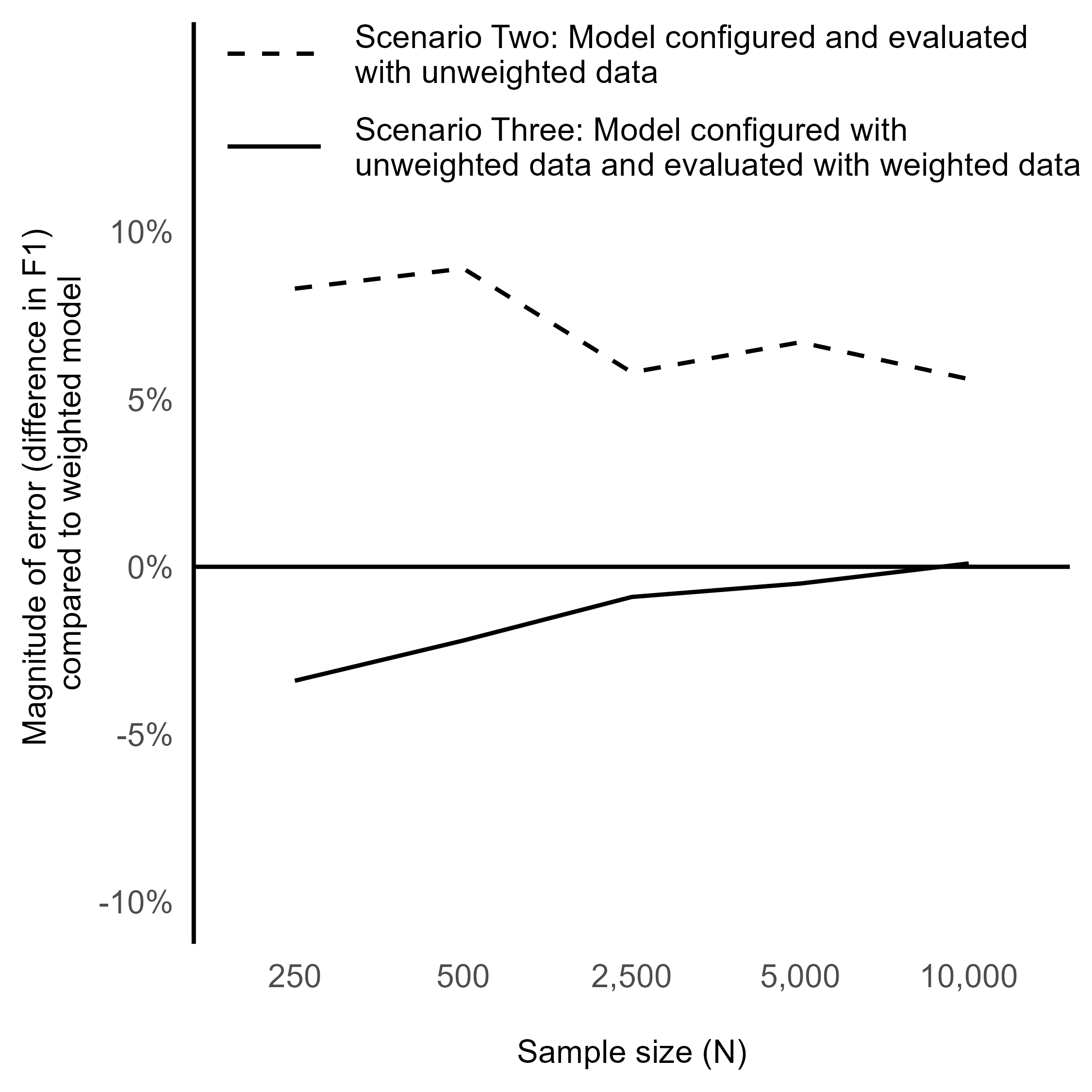

Supplement: S1 Fig — (TIF) [file pone.0280387.s001.tif]

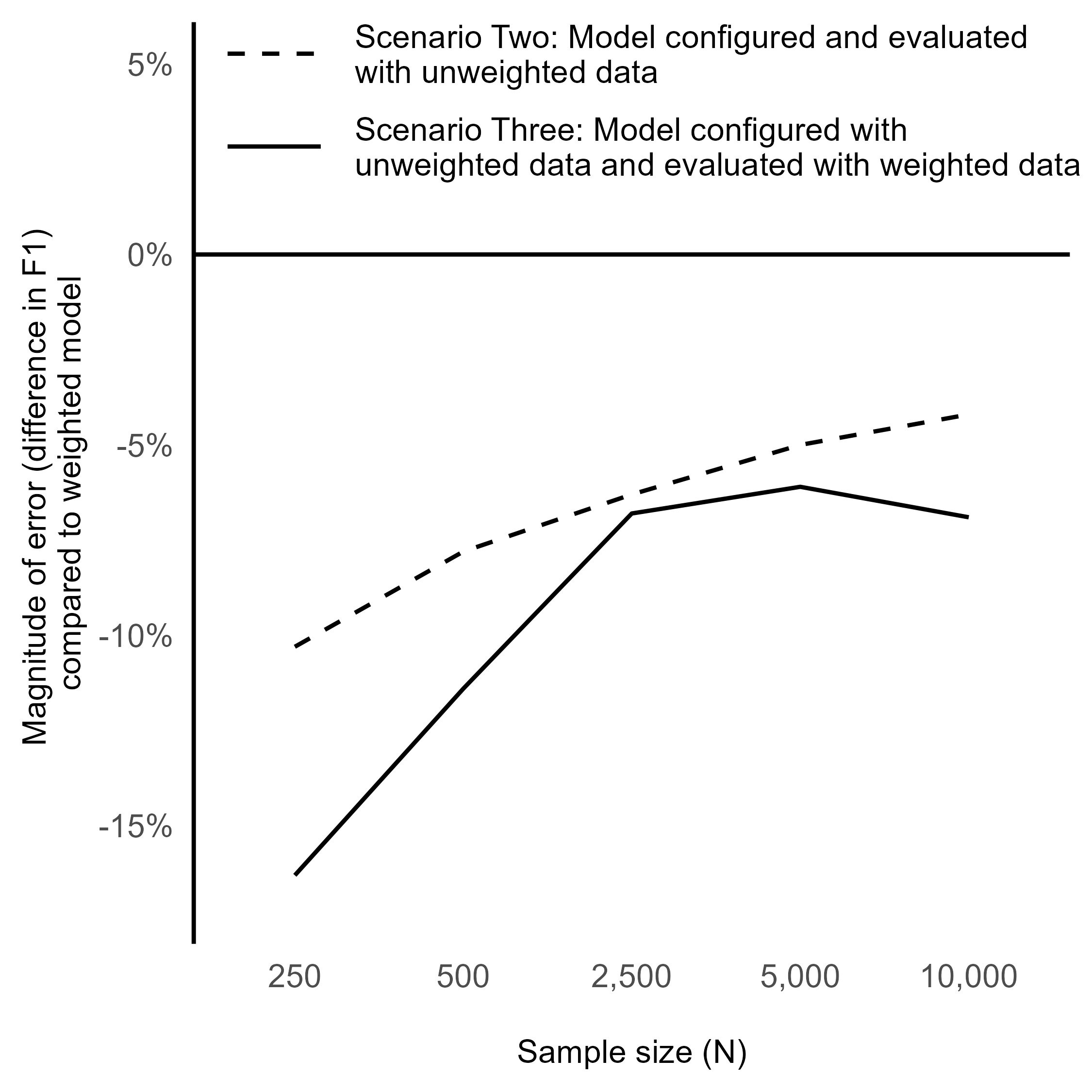

Supplement: S2 Fig — (TIF) [file pone.0280387.s002.tif]
