# Supplementary material for: Implementing machine learning methods with complex survey data: Lessons learned on the impacts of accounting sampling weights in gradient boosting
Source: PLoS One. 2023 Jan 13;18(1):e0280387. doi: 10.1371/journal.pone.0280387 (PMC9838837; doi:10.1371/journal.pone.0280387)
Supplement: S1 Table — (DOCX) [file pone.0280387.s003.docx]

**S1 Table. Description of case study and simulations^a^ performed, and final hyper-parameter sets for weighted models.**

| Sample Size | Weight variability^b^ | Predictor strength^c^ | Dimensionality^d^ | Observation (row) Subsample | Positive Class Weight | Lambda (Ridge Penalty) | Alpha (LASSO^e^ penalty) | N Trees | Min Child Weight | Max Tree Depth | Max Delta (Step Size) | Learn Rate | Gamma (Min Step Size) | Predictor (Col) Subsample |
| --- | --- | --- | --- | --- | --- | --- | --- | --- | --- | --- | --- | --- | --- | --- |
| **NHANES III** | | | | | | | | | | | | | | |
| 15,820 | n/a | n/a | 27 | 0.7 | 2 | 7 | 8 | 94 | 77 | 6 | 2 | 0.05 | 12 | 0.9 |
| **Baseline simulation ^f^** | | | | | | | | | | | | | | |
| 10,000 | 1 | 1 | 27 | 0.8 | 2 | 8 | 9 | 32 | 7 | 4 | 1 | 0.09 | 14 | 0.74 |
| 5,000 | 1 | 1 | 27 | 0.9 | 2 | 4 | 5 | 29 | 35 | 2 | 4 | 0.19 | 6 | 0.53 |
| 2,500 | 1 | 1 | 27 | 0.5 | 2 | 4 | 2 | 42 | 96 | 2 | 4 | 0.1 | 0 | 0.31 |
| 500 | 1 | 1 | 27 | 0.5 | 2 | 3 | 3 | 26 | 3 | 5 | 8 | 0.19 | 7 | 0.97 |
| 250 | 1 | 1 | 27 | 0.5 | 1 | 3 | 8 | 25 | 85 | 10 | 8 | 0.11 | 3 | 0.78 |
| **High weight variability** | | | | | | | | | | | | | | |
| 10,000 | 2 | 1 | 27 | 0.8 | 2 | 4 | 0 | 27 | 99 | 2 | 3 | 0.13 | 1 | 0.17 |
| 5,000 | 2 | 1 | 27 | 0.5 | 2 | 1 | 2 | 21 | 99 | 1 | 7 | 0.19 | 18 | 0.22 |
| 2,500 | 2 | 1 | 27 | 0.6 | 2 | 6 | 4 | 62 | 1 | 1 | 0 | 0.18 | 16 | 0.85 |
| 500 | 2 | 1 | 27 | 0.9 | 1 | 1 | 8 | 47 | 5 | 19 | 4 | 0.16 | 12 | 0.84 |
| 250 | 2 | 1 | 27 | 0.5 | 1 | 3 | 4 | 60 | 26 | 3 | 0 | 0.13 | 10 | 0.53 |
| **Low weight variability** | | | | | | | | | | | | | | |
| 10,000 | 0.5 | 1 | 27 | 0.9 | 2 | 6 | 0 | 96 | 44 | 1 | 4 | 0.11 | 2 | 0.33 |
| 5,000 | 0.5 | 1 | 27 | 0.7 | 2 | 3 | 5 | 76 | 65 | 2 | 7 | 0.07 | 2 | 0.2 |
| 2,500 | 0.5 | 1 | 27 | 0.6 | 1 | 3 | 9 | 73 | 65 | 1 | 9 | 0.19 | 4 | 0.67 |
| 500 | 0.5 | 1 | 27 | 0.7 | 2 | 9 | 0 | 16 | 84 | 5 | 2 | 0.07 | 5 | 0.84 |
| 250 | 0.5 | 1 | 27 | 0.8 | 2 | 5 | 5 | 97 | 41 | 2 | 2 | 0.15 | 7 | 0.89 |
| **Strong marginal predictors** | | | | | | | | | | | | | | |
| 10,000 | 1 | 2 | 27 | 0.7 | 2 | 7 | 5 | 81 | 38 | 2 | 8 | 0.18 | 1 | 0.85 |
| 5,000 | 1 | 2 | 27 | 0.8 | 2 | 1 | 6 | 80 | 99 | 2 | 3 | 0.14 | 0 | 0.95 |
| 2,500 | 1 | 2 | 27 | 0.5 | 1 | 3 | 0 | 42 | 17 | 1 | 4 | 0.17 | 19 | 0.75 |
| 500 | 1 | 2 | 27 | 0.5 | 2 | 6 | 0 | 55 | 86 | 14 | 7 | 0.15 | 18 | 0.96 |
| 250 | 1 | 2 | 27 | 0.7 | 2 | 9 | 5 | 92 | 20 | 6 | 9 | 0.17 | 9 | 0.54 |
| **Weak marginal predictors** | | | | | | | | | | | | | | |
| 10,000 | 1 | 0.5 | 27 | 0.5 | 2 | 6 | 1 | 40 | 49 | 1 | 5 | 0.19 | 9 | 0.79 |
| 5,000 | 1 | 0.5 | 27 | 0.6 | 2 | 6 | 5 | 29 | 50 | 1 | 1 | 0.19 | 0 | 0.43 |
| 2,500 | 1 | 0.5 | 27 | 0.7 | 2 | 3 | 2 | 16 | 36 | 1 | 3 | 0.19 | 2 | 0.71 |
| 500 | 1 | 0.5 | 27 | 0.6 | 2 | 7 | 3 | 20 | 63 | 7 | 8 | 0.18 | 1 | 0.7 |
| 250 | 1 | 0.5 | 27 | 0.7 | 1 | 3 | 0 | 15 | 84 | 1 | 6 | 0.01 | 17 | 0.91 |
| **Fewer marginal predictors** | | | | | | | | | | | | | | |
| 10,000 | 1 | 1,0 | 10 | 0.7 | 2 | 5 | 8 | 25 | 40 | 4 | 5 | 0.09 | 4 | 0.91 |
| 5,000 | 1 | 1,0 | 10 | 0.7 | 2 | 6 | 5 | 29 | 76 | 5 | 6 | 0.01 | 10 | 0.66 |
| 2,500 | 1 | 1,0 | 10 | 0.9 | 2 | 7 | 4 | 59 | 61 | 3 | 9 | 0.15 | 4 | 0.75 |
| 500 | 1 | 1,0 | 10 | 0.5 | 1 | 5 | 5 | 21 | 12 | 22 | 9 | 0.13 | 11 | 0.94 |
| 250 | 1 | 1,0 | 10 | 0.5 | 2 | 1 | 4 | 41 | 68 | 1 | 0 | 0.07 | 4 | 0.76 |
| **Null model** | | | | | | | | | | | | | | |
| 10,000 | 1 | 0 | 0 | 0.5 | 1 | 3 | 0 | 2 | 25 | 22 | 2 | 0.12 | 10 | 0.4 |
| 5,000 | 1 | 0 | 0 | 0.6 | 1 | 8 | 6 | 2 | 63 | 23 | 4 | 0.15 | 1 | 0.63 |
| 2,500 | 1 | 0 | 0 | 0.6 | 2 | 9 | 7 | 2 | 65 | 10 | 0 | 0.08 | 17 | 0.33 |
| 500 | 1 | 0 | 0 | 0.6 | 1 | 7 | 7 | 2 | 76 | 18 | 8 | 0.12 | 2 | 0.54 |
| 250 | 1 | 0 | 0 | 0.8 | 1 | 7 | 1 | 6 | 80 | 12 | 6 | 0.17 | 4 | 0.5 |
| **Search space^g^** | | | | 0.5-1 | 1-2 | 1-10 | 0-10 | 1-100 | 1-100 | 1-25 | 0-10 | 0.01-0.2 | 0-20 | 0.15-1 |
| **Default hyper-parameter set** | | | | 1 | 1 | 1 | 0 | 100 | 1 | 6 | ∞ | 0.3 | 0 | 1 |

NHANES, National Health and Nutrition Examination Survey.

^a^ All simulations were created in SAS 9.4 (SAS Institute, Inc., Cary, North Carolina).

^b^ Multiplication coefficient applied to variance term in weight simulation formula relative to NHANES III variance.

^c^ Multiplication coefficient applied to β terms in outcome simulation relative to NHANES III parameter estimates.

^d^ Number of predictors in model.

^e^ LASSO: least absolute shrinkage and selection operator

^f^ Weight variability, predictor strength, and dimensionality relative to NHANES III data.

^g^ Search space increments were: learning rate = 0.01, row sample = 0.1, col sample = 0.01, other parameters = 1.
