# Supplementary material for: Implementing machine learning methods with complex survey data: Lessons learned on the impacts of accounting sampling weights in gradient boosting
Source: PLoS One. 2023 Jan 13;18(1):e0280387. doi: 10.1371/journal.pone.0280387 (PMC9838837; doi:10.1371/journal.pone.0280387)
Supplement: S2 Table — (DOCX) [file pone.0280387.s004.docx]

**S2 Table. Description of case study and simulations^a^ performed, and final hyper-parameter sets for unweighted models.**

| Sample Size | Weight variability^b^ | Predictor strength^c^ | Dimensionality^d^ | Observation (row) Subsample | Positive Class Weight | Lambda (Ridge Penalty) | Alpha (LASSO^e^ penalty) | N Trees | Min Child Weight | Max Tree Depth | Max Delta (Step Size) | Learn Rate | Gamma (Min Step Size) | Predictor (Col) Subsample |
| --- | --- | --- | --- | --- | --- | --- | --- | --- | --- | --- | --- | --- | --- | --- |
| **NHANES III** | | | | | | | | | | | | | | |
| 15,820 | n/a | n/a | 27 | 0.8 | 2 | 7 | 1 | 86 | 34 | 23 | 2 | 0.18 | 7 | 0.71 |
| **Baseline simulation ^f^** | | | | | | | | | | | | | | |
| 10,000 | 1 | 1 | 27 | 0.7 | 2 | 2 | 0 | 30 | 7 | 3 | 7 | 0.12 | 15 | 0.38 |
| 5,000 | 1 | 1 | 27 | 0.5 | 2 | 1 | 8 | 60 | 5 | 7 | 8 | 0.16 | 19 | 0.27 |
| 2,500 | 1 | 1 | 27 | 0.5 | 1 | 8 | 1 | 94 | 21 | 7 | 3 | 0.16 | 2 | 0.81 |
| 500 | 1 | 1 | 27 | 0.7 | 1 | 6 | 6 | 41 | 20 | 18 | 3 | 0.15 | 17 | 0.28 |
| 250 | 1 | 1 | 27 | 0.6 | 2 | 8 | 0 | 31 | 10 | 23 | 7 | 0.05 | 1 | 0.34 |
| **High weight variability** | | | | | | | | | | | | | | |
| 10,000 | 2 | 1 | 27 | 0.6 | 2 | 4 | 1 | 61 | 4 | 2 | 8 | 0.18 | 0 | 0.61 |
| 5,000 | 2 | 1 | 27 | 0.6 | 2 | 2 | 0 | 87 | 36 | 8 | 4 | 0.14 | 15 | 0.15 |
| 2,500 | 2 | 1 | 27 | 0.9 | 2 | 4 | 0 | 56 | 46 | 13 | 9 | 0.11 | 5 | 0.45 |
| 500 | 2 | 1 | 27 | 0.8 | 1 | 8 | 0 | 93 | 19 | 8 | 8 | 0.18 | 2 | 0.42 |
| 250 | 2 | 1 | 27 | 0.6 | 2 | 8 | 4 | 20 | 1 | 21 | 5 | 0.19 | 1 | 0.27 |
| **Low weight variability** | | | | | | | | | | | | | | |
| 10,000 | 0.5 | 1 | 27 | 0.5 | 2 | 8 | 0 | 52 | 35 | 6 | 2 | 0.19 | 17 | 0.24 |
| 5,000 | 0.5 | 1 | 27 | 0.7 | 1 | 2 | 8 | 96 | 26 | 2 | 8 | 0.15 | 18 | 0.84 |
| 2,500 | 0.5 | 1 | 27 | 0.6 | 1 | 2 | 3 | 70 | 28 | 12 | 9 | 0.14 | 7 | 0.93 |
| 500 | 0.5 | 1 | 27 | 0.7 | 2 | 7 | 8 | 15 | 26 | 12 | 9 | 0.09 | 13 | 0.62 |
| 250 | 0.5 | 1 | 27 | 0.9 | 2 | 5 | 2 | 76 | 1 | 10 | 7 | 0.12 | 2 | 0.48 |
| **Strong marginal predictors** | | | | | | | | | | | | | | |
| 10,000 | 1 | 2 | 27 | 0.6 | 1 | 7 | 0 | 95 | 7 | 3 | 9 | 0.17 | 6 | 0.51 |
| 5,000 | 1 | 2 | 27 | 0.5 | 2 | 7 | 1 | 96 | 2 | 12 | 1 | 0.18 | 5 | 0.66 |
| 2,500 | 1 | 2 | 27 | 0.7 | 1 | 1 | 2 | 87 | 4 | 2 | 4 | 0.18 | 6 | 0.44 |
| 500 | 1 | 2 | 27 | 0.9 | 2 | 4 | 0 | 50 | 9 | 13 | 0 | 0.16 | 2 | 0.25 |
| 250 | 1 | 2 | 27 | 0.6 | 1 | 1 | 2 | 46 | 1 | 7 | 9 | 0.12 | 16 | 0.29 |
| **Weak marginal predictors** | | | | | | | | | | | | | | |
| 10,000 | 1 | 0.5 | 27 | 0.9 | 2 | 7 | 2 | 94 | 1 | 2 | 4 | 0.18 | 15 | 0.17 |
| 5,000 | 1 | 0.5 | 27 | 0.8 | 2 | 6 | 2 | 15 | 62 | 12 | 0 | 0.03 | 2 | 0.42 |
| 2,500 | 1 | 0.5 | 27 | 0.5 | 2 | 4 | 7 | 62 | 8 | 23 | 6 | 0.19 | 14 | 0.29 |
| 500 | 1 | 0.5 | 27 | 0.6 | 2 | 1 | 0 | 18 | 12 | 18 | 9 | 0.19 | 7 | 0.67 |
| 250 | 1 | 0.5 | 27 | 0.8 | 2 | 2 | 0 | 96 | 3 | 17 | 2 | 0.15 | 13 | 0.79 |
| **Fewer marginal predictors** | | | | | | | | | | | | | | |
| 10,000 | 1 | 1,0 | 10 | 0.5 | 2 | 6 | 0 | 1 | 10 | 15 | 5 | 0.03 | 2 | 0.46 |
| 5,000 | 1 | 1,0 | 10 | 0.6 | 2 | 3 | 2 | 80 | 31 | 21 | 0 | 0.19 | 1 | 0.98 |
| 2,500 | 1 | 1,0 | 10 | 0.5 | 2 | 1 | 1 | 2 | 1 | 21 | 4 | 0.12 | 3 | 0.89 |
| 500 | 1 | 1,0 | 10 | 0.6 | 2 | 2 | 2 | 54 | 6 | 18 | 1 | 0.18 | 5 | 0.83 |
| 250 | 1 | 1,0 | 10 | 0.7 | 2 | 5 | 6 | 93 | 4 | 16 | 0 | 0.16 | 0 | 0.51 |
| **Null model** | | | | | | | | | | | | | | |
| 10,000 | 1 | 0 | 0 | 0.5 | 2 | 6 | 0 | 1 | 10 | 15 | 5 | 0.03 | 2 | 0.46 |
| 5,000 | 1 | 0 | 0 | 0.6 | 2 | 3 | 2 | 80 | 31 | 21 | 0 | 0.19 | 1 | 0.98 |
| 2,500 | 1 | 0 | 0 | 0.5 | 2 | 1 | 1 | 2 | 1 | 21 | 4 | 0.12 | 3 | 0.89 |
| 500 | 1 | 0 | 0 | 0.6 | 2 | 2 | 2 | 54 | 6 | 18 | 1 | 0.18 | 5 | 0.83 |
| 250 | 1 | 0 | 0 | 0.7 | 2 | 5 | 6 | 93 | 4 | 16 | 0 | 0.16 | 0 | 0.51 |
| **Search space^g^** | | | | 0.5-1 | 1-2 | 1-10 | 0-10 | 1-100 | 1-100 | 1-25 | 0-10 | 0.01-0.2 | 0-20 | 0.15-1 |
| **Default hyper-parameter set** | | | | 1 | 1 | 1 | 0 | 100 | 1 | 6 | ∞ | 0.3 | 0 | 1 |

NHANES, National Health and Nutrition Examination Survey.

^a^ All simulations were created in SAS 9.4 (SAS Institute, Inc., Cary, North Carolina).

^b^ Multiplication coefficient applied to variance term in weight simulation formula relative to NHANES III variance.

^c^ Multiplication coefficient applied to β terms in outcome simulation relative to NHANES III parameter estimates.

^d^ Number of predictors in model.

^e^ LASSO: least absolute shrinkage and selection operator

^f^ Weight variability, predictor strength, and dimensionality relative to NHANES III data.

^g^ Search space increments were: learning rate = 0.01, row sample = 0.1, col sample = 0.01, other parameters = 1.
