# Supplementary material for: Implementing machine learning methods with complex survey data: Lessons learned on the impacts of accounting sampling weights in gradient boosting
Source: PLoS One. 2023 Jan 13;18(1):e0280387. doi: 10.1371/journal.pone.0280387 (PMC9838837; doi:10.1371/journal.pone.0280387)
Supplement: S3 Table — (DOCX) [file pone.0280387.s005.docx]

**S3 Table. Performance of gradient boosting models configured and evaluated under different design scenarios.**

|  |  | F1 Score  (95% CI) | |  | F1 Score (95% CI) |  |
| --- | --- | --- | --- | --- | --- | --- |
| N | Study design feature | Scenario One^a^  (gold standard) | Scenario Two^b^ | Error^d,f^ | Scenario Three^c^ | Error^e,f^ |
| 15,820 | NHANES III | 0.774 (0.761, 0.786) | 0.819 (0.812, 0.827) | 0.046 | 0.770 (0.757, 0.784) | -0.003 |
| 10,000 | Larger sample size | 0.725 (0.705, 0.745) | 0.798 (0.787, 0.809) | 0.073 | 0.722 (0.702, 0.742) | -0.004 |
| 5,000 | Medium sample size | 0.744 (0.717, 0.770) | 0.803 (0.787, 0.818) | 0.059 | 0.741 (0.716, 0.768) | -0.003 |
| 2,500 | Smaller sample size | 0.744 (0.704, 0.781) | 0.804 (0.782, 0.825) | 0.060 | 0.721 (0.677, 0.762) | -0.023 |
| 500 | Tiny sample | 0.818 (0.740, 0.880) | 0.846 (0.795, 0.888) | 0.028 | 0.787 (0.698, 0.856) | -0.031 |
| 250 | Extra tiny sample | 0.787 (0.675, 0.889) | 0.836 (0.766, 0.893) | 0.049 | 0.732 (0.625, 0.832) | -0.055 |
| 10,000 | High variability in weights | 0.806 (0.779, 0.828) | 0.791 (0.780, 0.802) | -0.015 | 0.800 (0.772, 0.824) | -0.005 |
| 5,000 | High variability in weights | 0.763 (0.713, 0.806) | 0.794 (0.778, 0.810) | 0.031 | 0.740 (0.683, 0.795) | -0.023 |
| 2,500 | High variability in weights | 0.845 (0.798, 0.875) | 0.82 (0.797, 0.839) | -0.024 | 0.833 (0.786, 0.866) | -0.012 |
| 500 | High variability in weights | 0.844 (0.705, 0.895) | 0.833 (0.778, 0.873) | -0.010 | 0.779 (0.659, 0.892) | -0.065 |
| 250 | High variability in weights | 0.938 (0.843, 0.967) | 0.852 (0.775, 0.907) | -0.086 | 0.813 (0.684, 0.945) | -0.125 |
| 10,000 | Low variability in weights | 0.799 (0.786, 0.812) | 0.799 (0.788, 0.809) | -0.001 | 0.798 (0.785, 0.811) | -0.001 |
| 5,000 | Low variability in weights | 0.789 (0.769, 0.807) | 0.792 (0.775, 0.807) | 0.003 | 0.787 (0.769, 0.805) | -0.002 |
| 2,500 | Low variability in weights | 0.796 (0.767, 0.821) | 0.792 (0.768, 0.813) | -0.003 | 0.794 (0.767, 0.819) | -0.002 |
| 500 | Low variability in weights | 0.849 (0.798, 0.887) | 0.842 (0.795, 0.882) | -0.006 | 0.839 (0.777, 0.884) | -0.010 |
| 250 | Low variability in weights | 0.820 (0.722, 0.884) | 0.787 (0.705, 0.847) | -0.034 | 0.801 (0.709, 0.866) | -0.020 |
| 10,000 | Strong marginal predictors | 0.846 (0.830, 0.861) | 0.886 (0.877, 0.895) | 0.040 | 0.840 (0.822, 0.857) | -0.006 |
| 5,000 | Strong marginal predictors | 0.864 (0.843, 0.884) | 0.886 (0.874, 0.899) | 0.022 | 0.855 (0.831, 0.875) | -0.010 |
| 2,500 | Strong marginal predictors | 0.864 (0.828, 0.895) | 0.892 (0.873, 0.909) | 0.028 | 0.854 (0.817, 0.888) | -0.010 |
| 500 | Strong marginal predictors | 0.905 (0.857, 0.948) | 0.907 (0.864, 0.938) | 0.002 | 0.877 (0.808, 0.934) | -0.028 |
| 250 | Strong marginal predictors | 0.900 (0.826, 0.947) | 0.895 (0.837, 0.935) | -0.005 | 0.816 (0.702, 0.909) | -0.084 |
| 10,000 | Weak marginal predictors | 0.576 (0.556, 0.597) | 0.663 (0.650, 0.676) | 0.087 | 0.577 (0.556, 0.600) | 0.000 |
| 5,000 | Weak marginal predictors | 0.584 (0.553, 0.614) | 0.662 (0.644, 0.680) | 0.079 | 0.597 (0.569, 0.624) | 0.013 |
| 2,500 | Weak marginal predictors | 0.630 (0.587, 0.665) | 0.689 (0.663, 0.712) | 0.059 | 0.627 (0.586, 0.665) | -0.003 |
| 500 | Weak marginal predictors | 0.634 (0.540, 0.722) | 0.696 (0.637, 0.750) | 0.062 | 0.600 (0.512, 0.695) | -0.034 |
| 250 | Weak marginal predictors | 0.735 (0.614, 0.824) | 0.756 (0.684, 0.818) | 0.020 | 0.743 (0.630, 0.829) | 0.008 |
| 10,000 | Fewer marginal predictors (10) | 0.679 (0.657, 0.700) | 0.735 (0.721, 0.746) | 0.056 | 0.680 (0.658, 0.701) | 0.001 |
| 5,000 | Fewer marginal predictors (10) | 0.681 (0.647, 0.710) | 0.748 (0.731, 0.764) | 0.067 | 0.676 (0.645, 0.707) | -0.005 |
| 2,500 | Fewer marginal predictors (10) | 0.683 (0.642, 0.721) | 0.741 (0.716, 0.765) | 0.058 | 0.674 (0.626, 0.715) | -0.009 |
| 500 | Fewer marginal predictors (10) | 0.661 (0.576, 0.736) | 0.750 (0.689, 0.802) | 0.089 | 0.639 (0.553, 0.724) | -0.022 |
| 250 | Fewer marginal predictors (10) | 0.660 (0.519, 0.772) | 0.743 (0.658, 0.810) | 0.083 | 0.626 (0.495, 0.750) | -0.034 |
| 10,000 | No marginal predictors | 0.318 (0.288, 0.346) | 0.276 (0.261, 0.291) | -0.042 | 0.249 (0.223, 0.274) | -0.069 |
| 5,000 | No marginal predictors | 0.348 (0.310, 0.380) | 0.298 (0.276, 0.318) | -0.050 | 0.287 (0.252, 0.322) | -0.061 |
| 2,500 | No marginal predictors | 0.367 (0.317, 0.414) | 0.304 (0.275, 0.333) | -0.063 | 0.300 (0.258, 0.344) | -0.068 |
| 500 | No marginal predictors | 0.436 (0.291, 0.540) | 0.358 (0.278, 0.431) | -0.078 | 0.322 (0.226, 0.407) | -0.114 |
| 250 | No marginal predictors | 0.498 (0.325, 0.603) | 0.394 (0.296, 0.475) | -0.103 | 0.335 (0.211, 0.451) | -0.163 |

NHANES III, National Health and Nutrition Examination Survey III; CI, confidence interval.

^a^ Scenario One: gradient boosting model configured and evaluated on weighted data (gold standard model).

^b^ Scenario Two: gradient boosting model configured and evaluated on unweighted data.

^c^ Scenario Three: gradient boosting model configured on unweighted data and evaluated on weighted data.

^d^ Difference in F1 score for Scenario Two compared to Scenario One.

^e^ Difference in F1 score for Scenario Three compared to Scenario One.

^f^ All displayed values were rounded to the 3^rd^ decimal place after errors were calculated from unrounded F1 scores. Displayed errors may therefore be nominally different than would be expected if calculated from the displayed F1 scores.
