# Supplementary material for: Implementing machine learning methods with complex survey data: Lessons learned on the impacts of accounting sampling weights in gradient boosting
Source: PLoS One. 2023 Jan 13;18(1):e0280387. doi: 10.1371/journal.pone.0280387 (PMC9838837; doi:10.1371/journal.pone.0280387)
Supplement: S4 Table — (DOCX) [file pone.0280387.s006.docx]

**S4 Table. Performance of gradient boosting models run with default hyper-parameters under different design scenarios.**

|  |  | F1 Score  (95% CI) | |  | F1 Score  (95% CI) |  |
| --- | --- | --- | --- | --- | --- | --- |
| N | Study design feature | Scenario One^a^  (gold standard) | Scenario Two^b^ | Error^d,f^ | Scenario Three^c^ | Error^e,f^ |
| 15,820 | NHANES III | 0.736 (0.723, 0.750) | 0.805 (0.798, 0.813) | 0.069 | 0.747 (0.732, 0.760) | 0.011 |
| 10,000 | Larger sample size | 0.608 (0.581, 0.631) | 0.735 (0.721, 0.748) | 0.127 | 0.639 (0.613, 0.664) | 0.031 |
| 5,000 | Medium sample size | 0.655 (0.623, 0.687) | 0.746 (0.728, 0.763) | 0.091 | 0.667 (0.633, 0.699) | 0.012 |
| 2,500 | Smaller sample size | 0.654 (0.605, 0.695) | 0.759 (0.735, 0.783) | 0.105 | 0.676 (0.631, 0.716) | 0.022 |
| 500 | Tiny sample | 0.736 (0.643, 0.804) | 0.785 (0.725, 0.838) | 0.050 | 0.741 (0.647, 0.816) | 0.006 |
| 250 | Extra tiny sample | 0.678 (0.548, 0.796) | 0.748 (0.649, 0.826) | 0.070 | 0.645 (0.524, 0.767) | 0.033 |
| 10,000 | High variability in weights | 0.695 (0.661, 0.727) | 0.733 (0.718, 0.745) | 0.038 | 0.738 (0.704, 0.765) | 0.043 |
| 5,000 | High variability in weights | 0.695 (0.644, 0.746) | 0.732 (0.713, 0.751) | 0.037 | 0.685 (0.623, 0.741) | 0.009 |
| 2,500 | High variability in weights | 0.764 (0.707, 0.803) | 0.769 (0.744, 0.792) | 0.005 | 0.805 (0.754, 0.841) | 0.041 |
| 500 | High variability in weights | 0.681 (0.552, 0.799) | 0.776 (0.715, 0.827) | 0.095 | 0.719 (0.586, 0.826) | 0.038 |
| 250 | High variability in weights | 0.56 (0.428, 0.757) | 0.825 (0.747, 0.883) | 0.265 | 0.820 (0.698, 0.948) | 0.260 |
| 10,000 | Low variability in weights | 0.715 (0.697, 0.729) | 0.727 (0.713, 0.740) | 0.013 | 0.723 (0.705, 0.740) | 0.009 |
| 5,000 | Low variability in weights | 0.723 (0.698, 0.745) | 0.734 (0.714, 0.753) | 0.011 | 0.722 (0.696, 0.745) | 0.001 |
| 2,500 | Low variability in weights | 0.746 (0.715, 0.775) | 0.720 (0.691, 0.745) | 0.025 | 0.722 (0.690, 0.753) | 0.023 |
| 500 | Low variability in weights | 0.796 (0.725, 0.849) | 0.774 (0.707, 0.823) | 0.022 | 0.773 (0.702, 0.828) | 0.024 |
| 250 | Low variability in weights | 0.712 (0.602, 0.790) | 0.705 (0.610, 0.781) | 0.007 | 0.718 (0.601, 0.805) | 0.007 |
| 10,000 | Strong marginal predictors | 0.810 (0.791, 0.827) | 0.856 (0.845, 0.865) | 0.045 | 0.803 (0.784, 0.821) | 0.008 |
| 5,000 | Strong marginal predictors | 0.816 (0.791, 0.841) | 0.857 (0.843, 0.872) | 0.041 | 0.813 (0.788, 0.837) | 0.003 |
| 2,500 | Strong marginal predictors | 0.815 (0.775, 0.851) | 0.867 (0.847, 0.885) | 0.052 | 0.812 (0.773, 0.849) | 0.003 |
| 500 | Strong marginal predictors | 0.825 (0.748, 0.888) | 0.888 (0.849, 0.921) | 0.064 | 0.878 (0.825, 0.925) | 0.053 |
| 250 | Strong marginal predictors | 0.733 (0.621, 0.829) | 0.859 (0.796, 0.908) | 0.126 | 0.811 (0.704, 0.892) | 0.078 |
| 10,000 | Weak marginal predictors | 0.430 (0.405, 0.453) | 0.557 (0.541, 0.572) | 0.127 | 0.447 (0.422, 0.471) | 0.017 |
| 5,000 | Weak marginal predictors | 0.441 (0.406, 0.472) | 0.557 (0.537, 0.578) | 0.117 | 0.452 (0.417, 0.485) | 0.011 |
| 2,500 | Weak marginal predictors | 0.512 (0.465, 0.557) | 0.601 (0.571, 0.627) | 0.090 | 0.509 (0.468, 0.551) | 0.003 |
| 500 | Weak marginal predictors | 0.519 (0.420, 0.609) | 0.573 (0.506, 0.633) | 0.054 | 0.537 (0.440, 0.621) | 0.018 |
| 250 | Weak marginal predictors | 0.498 (0.396, 0.593) | 0.587 (0.482, 0.669) | 0.090 | 0.448 (0.335, 0.564) | 0.050 |
| 10,000 | Fewer marginal predictors (10) | 0.560 (0.533, 0.584) | 0.635 (0.620, 0.650) | 0.055 | 0.563 (0.539, 0.588) | 0.073 |
| 5,000 | Fewer marginal predictors (10) | 0.580 (0.546, 0.613) | 0.670 (0.648, 0.691) | 0.039 | 0.579 (0.545, 0.615) | 0.077 |
| 2,500 | Fewer marginal predictors (10) | 0.604 (0.558, 0.649) | 0.682 (0.653, 0.710) | 0.042 | 0.588 (0.539, 0.641) | 0.060 |
| 500 | Fewer marginal predictors (10) | 0.542 (0.459, 0.625) | 0.683 (0.615, 0.742) | 0.071 | 0.589 (0.501, 0.671) | 0.022 |
| 250 | Fewer marginal predictors (10) | 0.360 (0.258, 0.485) | 0.547 (0.442, 0.645) | 0.146 | 0.397 (0.273, 0.523) | 0.088 |
| 10,000 | No marginal predictors | 0.144 (0.116, 0.171) | 0.089 (0.076, 0.102) | 0.076 | 0.071 (0.053, 0.091) | 0.004 |
| 5,000 | No marginal predictors | 0.181 (0.148, 0.213) | 0.142 (0.120, 0.164) | 0.090 | 0.104 (0.081, 0.129) | 0.001 |
| 2,500 | No marginal predictors | 0.180 (0.133, 0.227) | 0.139 (0.109, 0.166) | 0.078 | 0.120 (0.086, 0.163) | 0.016 |
| 500 | No marginal predictors | 0.129 (0.084, 0.244) | 0.200 (0.129, 0.281) | 0.141 | 0.152 (0.087, 0.249) | 0.047 |
| 250 | No marginal predictors | 0.141 (0.089, 0.255) | 0.287 (0.210, 0.388) | 0.187 | 0.229 (0.136, 0.344) | 0.037 |

NHANES III, National Health and Nutrition Examination Survey III; CI, confidence interval.

^a^ Scenario One: gradient boosting model configured and evaluated on weighted data (gold standard model).

^b^ Scenario Two: gradient boosting model configured and evaluated on unweighted data.

^c^ Scenario Three: gradient boosting model configured on unweighted data and evaluated on weighted data.

^d^ Difference in F1 score for Scenario Two compared to Scenario One.

^e^ Difference in F1 score for Scenario Three compared to Scenario One.

^f^ All displayed values were rounded to the 3^rd^ decimal place after errors were calculated from unrounded F1 scores. Displayed errors may therefore be nominally different than would be expected if calculated from the displayed F1 scores.
