# Supplementary material for: Implementing machine learning methods with complex survey data: Lessons learned on the impacts of accounting sampling weights in gradient boosting
Source: PLoS One. 2023 Jan 13;18(1):e0280387. doi: 10.1371/journal.pone.0280387 (PMC9838837; doi:10.1371/journal.pone.0280387)
Supplement: S1 Appendix — (DOCX) [file pone.0280387.s007.docx]

**S1 Appendix. Using F1 score as an evaluation metric**

We used the F1 score to evaluate model performance during the hyperparameter selection process. This statistic is calculated as the harmonic mean of sensitivity and positive predictive value:

$$F_{1}=\frac{1}{\left( \frac{1}{Sensitivity}+\frac{1}{Positive Predictive Value} \right)}$$

$$=\frac{2 \left( Sensitivity*Positive Predictive Value \right)}{\left( Sensitivity+Positive Predictive Value \right)}$$

$$=\frac{2* True Positives}{\left( 2*True Positives+False Positives+False Negatives \right)}$$

Where sensitivity (also known as recall) is the proportion of all true cases identified as cases by the algorithm and positive predictive value (also known as precision) is the probability that an observation identified as a case by the algorithm is truly a case [1]. By using the harmonic mean of these two measures, the algorithm weights both performance measures “equally” in evaluating the overall model performance, resulting in rarer conditions (cases in our example) being more weight individually [2]. Given a fixed number of cases in the sample, maximizing the score is achieved by minimizing the total number of false positives and false negatives.

**References**

1. Sokolova M, Lapalme G. A systematic analysis of performance measures for classification tasks. Information Processing & Management. 2009;45(4):427-37. doi: 10.1016/j.ipm.2009.03.002.

2. Lipton ZC, Elkan C, Narayanaswamy B. Thresholding classifiers to maximize F1 score. arXiv preprint arXiv:14021892. 2014. doi: 10.48550/arXiv.1402.1892.
