# Supplementary material for: Implementing machine learning methods with complex survey data: Lessons learned on the impacts of accounting sampling weights in gradient boosting
Source: PLoS One. 2023 Jan 13;18(1):e0280387. doi: 10.1371/journal.pone.0280387 (PMC9838837; doi:10.1371/journal.pone.0280387)
Supplement: S2 Appendix — (DOCX) [file pone.0280387.s008.docx]

**S2 Appendix. XGBoost hyperparameter interpretations and ranges used for hyperparameter sample space.**

| **Hyperparameter** | **Range Used** | **Description** |
| --- | --- | --- |
| Observation (row) subsample | 0.5-1 | Proportion of dataset sampled to use in fitting each sequential tree model. For a value of 1, the entire sample is used for each tree model. |
| Positive Class Weight | 1 or 2 | The weight applied to cases for calculating goodness-of-fit statistics during the tree growth process. A value of 1 weights cases and controls equally; a value of 2 gives cases twice as much weight to cases. |
| Lambda (Ridge Penalty) | 1-10 | A *square* penalty term added to the tree model that attempts to reduce model over-fit by worsening the apparent model fit statistic as the number and magnitude of coefficients in the model increases (analogous to lasso regression). |
| Alpha  (LASSO penalty) | 0-10 | A *linear* penalty term added to the tree model that attempts to reduce model over-fit by worsening the apparent model fit statistic as the number and magnitude of terms in the model increases (analogous to ridge regression). |
| N trees | 1-1000 | The total number of tree models used in the ensemble model (individual tree predictions are summed to determine the overall model prediction). |
| Min Child Weight | 1-100 | The minimum number of (weighted) observations needed in each terminal branch (leaf) to keep a split in an individual tree model. |
| Max Tree Depth | 1-25 | The maximum number of levels used in the tree model (each branch can split again at each level; there are 2^n-1^ possible terminal branches). Higher depth models additional interaction between predictors but increases the likelihood of memorizing the input data. |
| Max Delta Step  (step size) | 0-10 | Slows the branching within each level of each regression tree by limiting the influence of an individual split. Lower values result in more splits on key variables occurring later in the tree structure, leading to more interaction terms for important predictors. |
| Learning Rate | 0.01-0.2 | The degree to which predictions from sequential tree models contribute to the overall model prediction. Smaller values increase model diversity by reducing the impact of any one tree model on the final prediction. |
| Gamma (Min Step Size) | 0-20 | Limits the branching within each level of each regression tree by specifying the minimum level of improvement needed to a tree’s prediction to create a new split. |
| Predictor (Col) subsample | 0.15-1 | Proportion of predictor variables sampled to use in fitting each sequential tree model. For a value of 1, all predictors are used for all tree models. |
