# Supplementary material for: Implementing machine learning methods with complex survey data: Lessons learned on the impacts of accounting sampling weights in gradient boosting
Source: PLoS One. 2023 Jan 13;18(1):e0280387. doi: 10.1371/journal.pone.0280387 (PMC9838837; doi:10.1371/journal.pone.0280387)
Supplement: S1 Code — (PDF) [file pone.0280387.s009.pdf]

Create simulated dataset

In [1]:

```
### Example code script

# in this example code, we show the model fit procedure and evaluation
# metric calculation for a small hyperparameter search space and an
# example randomized dataset. In our main analysis, this code was
# parallelized on a computing cluster using slurm to test many
# hyperparameter combinations across many simulated datasets
# (each of which was based on NHANES data). For this example, we also
# simplified the process of identifying the best hyperparameter set
# among all potential sets tested (which was originally done in R).
```

In [2]:

```
### Setup

import pandas as pd
import numpy as np
from sklearn import datasets
import matplotlib.pyplot as plt
import xgboost as xgb
from sklearn.model_selection import RandomizedSearchCV, StratifiedKFold
from sklearn.metrics import log_loss, make_scorer, f1_score
from sklearn.dummy import DummyClassifier
from xgboost import XGBClassifier

# set the size of the synthetic dataset
n_vars = 10
n_rows = 10000
```

In [3]:

```
# Generate a linear regression problem

X_array, Y_numeric = datasets.make_regression(n_samples = n_rows, n_features = n_vars,
                                n_informative = n_vars, n_targets = 1,
                                noise = 0.0)

# Create a dichotomous Y
Y = ( Y_numeric > 0 ) * 1

# Create a data frame of X with variables x1...xn
X = pd.DataFrame(X_array, columns=['x'+str(i) for i in list(range(1,n_vars+1))])

# Create weights and "no_weights" vectors (used for unweighted models/calculations)
wt = np.random.uniform(low=0.5, high=1.5, size=n_rows)
no_wt = np.array([1] * n_rows)
```

In [4]:

```
# inspect X and an example of xi (x1)
print(X.columns)
plt.hist(X['x1'], bins=100)
plt.show()
```

```
Index(['x1', 'x2', 'x3', 'x4', 'x5', 'x6', 'x7', 'x8', 'x9', 'x10'], dtype='object')
```

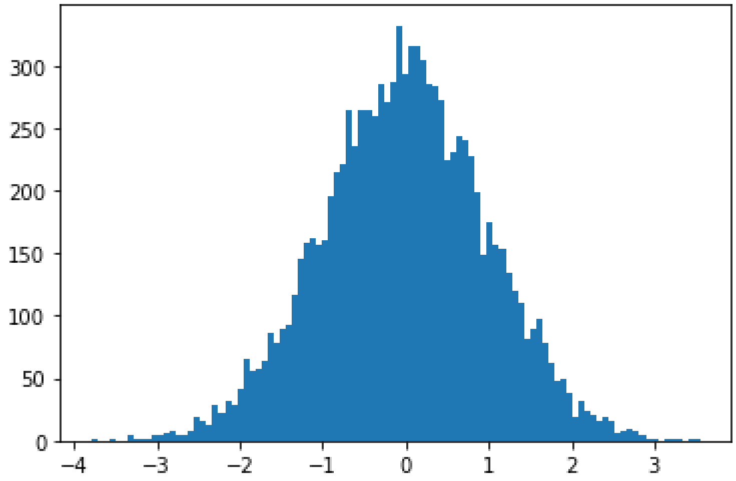

In [5]:

```
# inspect Y
print(type(Y))
plt.scatter(Y, Y_numeric)
plt.show()
```

```
<class 'numpy.ndarray'>
```

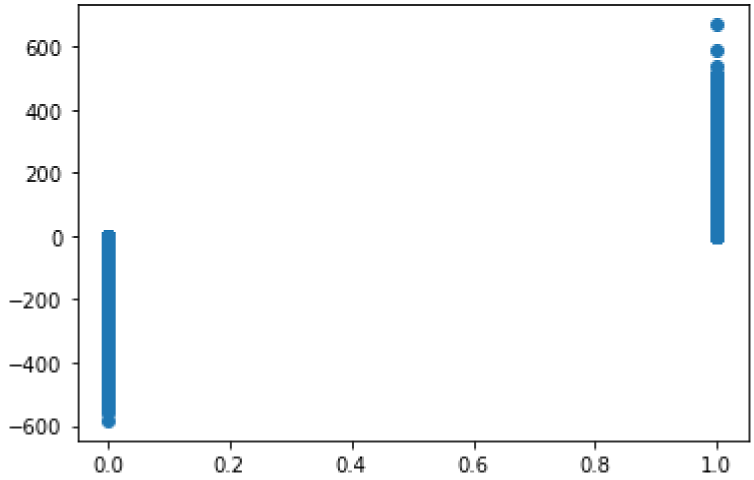

In [6]:

```
# inspect weights
print(type(wt))
plt.hist(wt)
plt.show()
```

```
<class 'numpy.ndarray'>
```

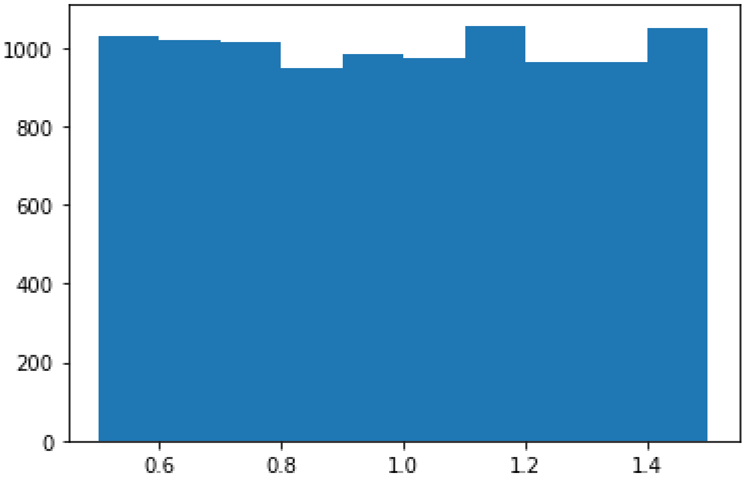

In [7]:

```
# inspect no_wt weights vector (all 1)
print(type(no_wt))
plt.hist(no_wt)
plt.show()
```

```
<class 'numpy.ndarray'>
```

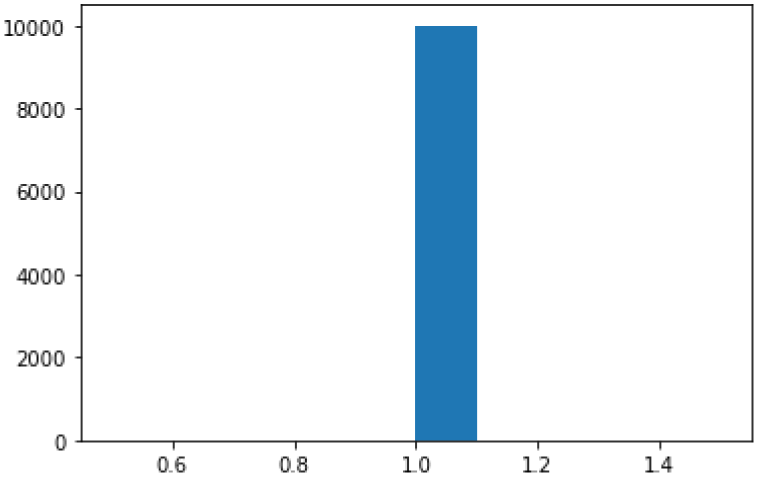

Configure Machine Learning Model

In [8]:

```
### Set hyperparameter search space
# max_depth and n_estimators are set to low values to enable
# running on a basic machine
hyperparameters = {
        #'learning_rate' :   np.arange(0.01, 0.2, 0.01),
        'max_depth' :       np.arange(1, 10, 1),
        'n_estimators' :    np.arange(1, 10, 1),
        #'gamma':            np.arange(0, 20, 1),
        #'min_child_weight': np.arange(1, 100, 1),
        #'max_delta_step':   np.arange(0, 10, 1),
        'subsample':        np.arange(0.5, 1, 0.1),
        #'reg_lambda':       np.arange(1, 10, 1),
        #'reg_alpha':        np.arange(0, 10, 1),
        'colsample_bytree': np.arange(0.5, 1, 0.1)
    }

hyperparameters
```

Out[8]:
```
{'max_depth': array([1, 2, 3, 4, 5, 6, 7, 8, 9]),
 'n_estimators': array([1, 2, 3, 4, 5, 6, 7, 8, 9]),
 'subsample': array([0.5, 0.6, 0.7, 0.8, 0.9]),
 'colsample_bytree': array([0.5, 0.6, 0.7, 0.8, 0.9])}
```

In [9]:
```
### set up weight objects to implement scoring strategy
weight_index = ['r%d' % x for x in range(len(Y))]
weight_frame = pd.DataFrame(wt, index=weight_index)
weight_frame_null = pd.DataFrame(no_wt, index=weight_index)
```

In [10]:
```
### set scoring strategy

# patch to the default f1 scorer to ensure that sample weights (or no weights)
# are read in cross-validation scoring

def score_f(y_true, y_pred, sample_weight):

    # dichotomize y
    y_bin = [1]*(y_pred > 0.5)

    # calculate f1 score using the sample weight pulled from the sample weight frame
    return f1_score(y_true, y_bin, sample_weight=sample_weight)

# test function on simple case (will produce f1 score near 0.5 with no information)
y_bad_predictions = np.random.uniform(low=0, high=1, size=len(Y))
score_f(y_true = Y, y_pred=y_bad_predictions, sample_weight=weight_frame)
```

Out[10]:
```
0.48587707109100087
```

In [11]:
```
### Define weighted and unweigted scorers

# Define weighted scorer
score_params_wt = {"sample_weight": wt}
f1_scorer_wt = make_scorer(score_f,
                           greater_is_better=True,
                           needs_proba=True,
                           needs_threshold=False,
                           **score_params_wt)      # pass weights parameters

# Define unweighted scorer
score_params_no_wt = {"sample_weight": no_wt}
f1_scorer_no_wt = make_scorer(score_f,
                           greater_is_better=True,
                           needs_proba=True,
                           needs_threshold=False,
                           **score_params_no_wt)   # no pass no weights parameters
```

In [12]:
```
# inspect calls
print(f1_scorer_wt)
print(f1_scorer_no_wt)
```

```
make_scorer(score_f, needs_proba=True, sample_weight=[0.73818714 0.59403749 0.84229755
... 1.25344446 1.36647885 1.31031648])
make_scorer(score_f, needs_proba=True, sample_weight=[1 1 1 ... 1 1 1])
```

In [13]:
```
# define the approach and objective
learner = xgb.XGBClassifier(objective='binary:logistic', use_label_encoder=False,
                            eval_metric='logloss')

learner
```

Out[13]:
```
XGBClassifier(base_score=None, booster=None, colsample_bylevel=None,
              colsample_bynode=None, colsample_bytree=None,
              enable_categorical=False, eval_metric='logloss', gamma=None,
              gpu_id=None, importance_type=None, interaction_constraints=None,
              learning_rate=None, max_delta_step=None, max_depth=None,
              min_child_weight=None, missing=nan, monotone_constraints=None,
              n_estimators=100, n_jobs=None, num_parallel_tree=None,
              predictor=None, random_state=None, reg_alpha=None,
              reg_lambda=None, scale_pos_weight=None, subsample=None,
              tree_method=None, use_label_encoder=False,
              validate_parameters=None, verbosity=None)
```

In [14]:
```
# combine configuration into a single object to define the model and fit process
# we need to create two objects (for weighted/unweighted)
# that will be modified with the fit method
search_config_wt = RandomizedSearchCV(learner, param_distributions=hyperparameters,
                                      scoring='f1',
                                      cv=5, verbose=0, n_jobs=4, n_iter=16, refit=False)
search_config_no_wt = RandomizedSearchCV(learner, param_distributions=hyperparameters,
                                      scoring='f1',
                                      cv=5, verbose=0, n_jobs=4, n_iter=16, refit=True)
```

In [15]:
```
# fit models to data using model configurations
search_result_wt = search_config_wt.fit(X_array, Y, sample_weight=wt)
search_result_no_wt = search_config_no_wt.fit(X_array, Y, sample_weight=no_wt)
```

In [16]:
```
# extract summaries for weighted model fit
search_output_wt = pd.DataFrame.from_dict(search_result_wt.cv_results_)

# inspect best hyperparameters
search_output_wt.sort_values(by=['rank_test_score'])
```

Out[16]:

	mean_fit_time	std_fit_time	mean_score_time	std_score_time	param_subsample	param_n_estimators
**14**	0.068574	0.019527	0.007330	0.003750	0.8	9
**8**	0.120689	0.004168	0.002377	0.002180	0.9	9
**11**	0.087066	0.008208	0.002560	0.003792	0.7	5
**9**	0.061259	0.003826	0.006329	0.003951	0.5	5

	mean_fit_time	std_fit_time	mean_score_time	std_score_time	param_subsample	param_n_estimators
**6**	0.051323	0.008182	0.006122	0.001575	0.6	5
**12**	0.036050	0.004830	0.004009	0.004910	0.6	3
**7**	0.050426	0.004668	0.004007	0.004201	0.7	2
**3**	0.034751	0.003267	0.005751	0.000664	0.5	3
**4**	0.039672	0.007489	0.006295	0.002628	0.8	1
**15**	0.026133	0.009125	0.002406	0.003887	0.9	4
**0**	0.037462	0.010979	0.005208	0.000820	0.8	5
**10**	0.022444	0.003860	0.004024	0.004928	0.6	3
**2**	0.023129	0.005914	0.004451	0.004688	0.6	5
**1**	0.017643	0.001615	0.003179	0.001896	0.5	2
**5**	0.024459	0.004863	0.002607	0.001708	0.5	4
**13**	0.020149	0.000117	0.004433	0.004667	0.5	5

In [17]:

```
# inspect unweighted models
search_output_no_wt = pd.DataFrame.from_dict(search_result_no_wt.cv_results_)
search_output_no_wt.sort_values(by=['rank_test_score'])
```

Out[17]:

	mean_fit_time	std_fit_time	mean_score_time	std_score_time	param_subsample	param_n_estimators
**4**	0.122424	0.003143	0.006420	0.004468	0.8	9

	mean_fit_time	std_fit_time	mean_score_time	std_score_time	param_subsample	param_n_estimators
**9**	0.065442	0.004394	0.006414	0.003672	0.6	7
**11**	0.071011	0.003627	0.001193	0.000974	0.9	5
**0**	0.059747	0.000792	0.000806	0.000987	0.8	5
**15**	0.061967	0.011560	0.004514	0.003985	0.7	4
**5**	0.036521	0.004439	0.007215	0.003718	0.5	3
**1**	0.076490	0.004761	0.008719	0.000879	0.8	5
**13**	0.046521	0.006570	0.006095	0.004341	0.7	7
**14**	0.036088	0.004727	0.004008	0.004908	0.8	4
**7**	0.044184	0.004326	0.004436	0.004671	0.7	3
**8**	0.023456	0.004118	0.004616	0.004466	0.5	3
**10**	0.021110	0.003599	0.005475	0.004085	0.7	2
**3**	0.020023	0.000243	0.002004	0.004007	0.6	1
**2**	0.023166	0.003817	0.006651	0.003669	0.9	4
**6**	0.013269	0.003539	0.004809	0.003926	0.5	1
**12**	0.015066	0.004120	0.000000	0.000000	0.7	2

In [18]:
```python
# inspect distribution of model fit scores
_ = plt.hist(search_output_wt['mean_test_score'])
```

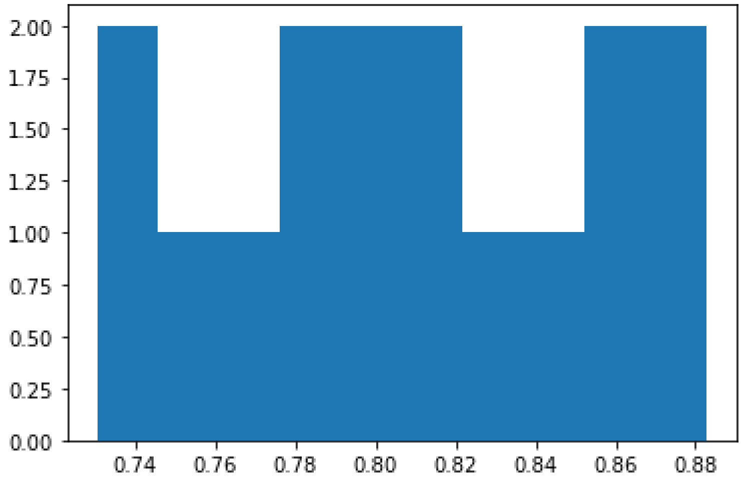

In [19]:
```python
# inspect model output as a function of hyperparameter
# in this example, an increasing the number of estimators (trees)
# improves the fit score
_ = plt.scatter(search_output_wt['param_n_estimators'],
                search_output_wt['mean_test_score'])
```

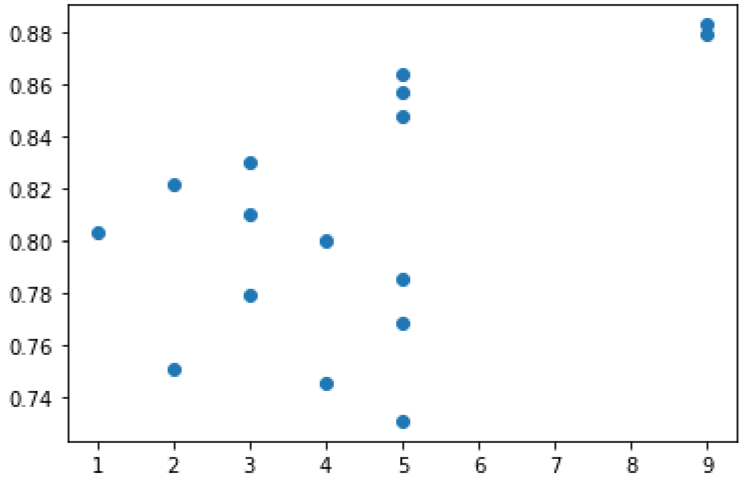

Estimate predicted probabilities

Refit best model to get predicted probabilities needed to calculate performance measures.

In [20]:
```python
# get best hyperparameters fopr weighted models results of search space
best_hyperparameters_wt = search_output_wt.loc[search_output_wt['rank_test_score']==1,
                                               search_output_wt.columns.str.contains('param_')]

# remove param_ to match xgboost parameter names
best_hyperparameters_wt.columns = \
  best_hyperparameters_wt.columns.str.replace("param_", "")

# convert to dictionary for xgboost
best_hyperparameters_wt = \
  best_hyperparameters_wt.to_dict(orient='list')
```

```
# parse refit params (strip arrays from dictionary)
for k, v in best_hyperparameters_wt.items():
    best_hyperparameters_wt[k] = best_hyperparameters_wt[k][0]

best_hyperparameters_wt
```

Out[20]:
```
{'subsample': 0.7999999999999999,
 'n_estimators': 9,
 'max_depth': 5,
 'colsample_bytree': 0.6}
```

In [21]:
```
# get best hyperparameters for unweighted models from results of search space
best_hyperparameters_no_wt = \
  search_output_no_wt.loc[search_output_no_wt['rank_test_score']==1,
  search_output_no_wt.columns.str.contains('param_')]

# remove param_ to match xgboost parameter names
best_hyperparameters_no_wt.columns = \
  best_hyperparameters_no_wt.columns.str.replace("param_", "")

# convert to dictionary for xgboost
best_hyperparameters_no_wt = best_hyperparameters_no_wt.to_dict(orient='list')

# parse refit params (strip arrays from dictionary)
for k, v in best_hyperparameters_no_wt.items():
    best_hyperparameters_no_wt[k] = best_hyperparameters_no_wt[k][0]

best_hyperparameters_no_wt
```

Out[21]:
```
{'subsample': 0.7999999999999999,
 'n_estimators': 9,
 'max_depth': 8,
 'colsample_bytree': 0.6}
```

In [22]:
```
def cv_refit(refit_params, use_weights):
    # calculuates cv prediction scores instead of full dataset refit prediction scores

    # set weights for model based on use_weights argument (True/False)
    if use_weights==False:
        model_wt = no_wt
    else:
        model_wt = wt

    # define split strategy (same as used in original fit)
    skf = StratifiedKFold(n_splits=5)

    # create empty arrays to store fold data
    y_actual = np.array([])
    y_predicted = np.array([])
    split_index = np.array([])
    orig_wt = np.array([])
    fit_wt = np.array([])

    # iterator for split id
    split_i = 1

    # loop through each split
    for train_index, test_index in skf.split(X_array, Y):
```

```
        #print(Y)
        #print(test_index)
        #print(Y[train_index])
        #print(Y[test_index])

        # extract observations by index
        X_train, X_test = X_array[train_index], X_array[test_index]
        y_train, y_test = Y[train_index], Y[test_index]
        wt_train, wt_test = model_wt[train_index], model_wt[test_index]

        # refit on data subset with refit parameters
        learner = XGBClassifier(objective='binary:logistic',
                                use_label_encoder=False, **refit_params,
                                eval_metric='logloss')
        learner.fit(X_train, y_train, sample_weight=wt_train)

        # build predictions and extract y=1 probabilities
        y_pred = learner.predict_proba(X_test)[:,1]

        # append predictions to each storage array
        y_predicted = np.append(y_predicted, y_pred)
        y_actual = np.append(y_actual, y_test)
        split_index = np.append(split_index, [split_i]*len(y_pred))

        # increment split id
        split_i = split_i+1

    # wrap variables together
    output = pd.DataFrame({'y_predicted':y_predicted, 'y_actual':y_actual,
                           'split':split_index})

    return(output)
```

In [23]:

```
predictions_wt = cv_refit(best_hyperparameters_wt, use_weights=True)
predictions_no_wt = cv_refit(best_hyperparameters_wt, use_weights=False)
```

In [24]:

```
predictions_wt
```

Out[24]:

	y_predicted	y_actual	split
**0**	0.632279	1.0	1.0
**1**	0.144563	0.0	1.0
**2**	0.827994	1.0	1.0
**3**	0.240502	0.0	1.0
**4**	0.174167	0.0	1.0
**...**	...	...	...
**9995**	0.716244	1.0	5.0
**9996**	0.565513	0.0	5.0
**9997**	0.875227	1.0	5.0

	y_predicted	y_actual	split
**9998**	0.853681	1.0	5.0
**9999**	0.885691	1.0	5.0

10000 rows × 3 columns

In [25]:

```
predictions_no_wt
```

Out[25]:

	y_predicted	y_actual	split
**0**	0.778868	1.0	1.0
**1**	0.215422	0.0	1.0
**2**	0.880617	1.0	1.0
**3**	0.211611	0.0	1.0
**4**	0.220783	0.0	1.0
**...**	...	...	...
**9995**	0.766550	1.0	5.0
**9996**	0.375083	0.0	5.0
**9997**	0.909065	1.0	5.0
**9998**	0.857344	1.0	5.0
**9999**	0.864226	1.0	5.0

10000 rows × 3 columns

Calculate performance metrics

In [26]:

```
# define function to calculate weights
def scenario_f1(pred, w):

    # pred      predicted probability dataset
    # w         weights set (wt or no_wt)

    # get test folds used in the original model fit
    test_folds = []
    for train,test in StratifiedKFold().split(X, Y):
        test_folds.append(test)

    scenario = []

    for test_set in test_folds:

        test_y = pred['y_actual'][test_set]
        test_y_pred = pred['y_predicted'][test_set] > 0.5
        test_wt    = w[test_set]

        scenario.append(f1_score(y_true = test_y,
```

```python
                                      y_pred = test_y_pred,
                                      sample_weight = test_wt))

    # return average score across 5 folds
    print(scenario)

    return np.array(scenario).mean()
```

In [27]:

```python
# Scenario 1: Weights used in model fitting and in used in evaluating f1 score
scenario_f1(pred = predictions_wt, w = wt)
```

```
[0.8792598110486083, 0.8779998646760891, 0.8867651341307285, 0.8853441029925988, 0.87870
55308578736]
```

Out[27]: 0.8816148887411798

In [28]:

```python
# Scenario 2: No weights used in model fitting or in evalauating f1 score
scenario_f1(pred = predictions_no_wt, w = no_wt)
```

```
[0.8916750250752257, 0.8882205513784461, 0.8835820895522388, 0.8829098156452416, 0.89101
33843212237]
```

Out[28]: 0.8874801731944751

In [29]:

```python
# Scenario 3: No weights used in model fitting, weights used in evaluating score
scenario_f1(pred = predictions_no_wt, w = wt)
```

```
[0.884923222775337, 0.8838723141190269, 0.8818610835708355, 0.8865307108654402, 0.893009
379481635]
```

Out[29]: 0.8860393421624548
